# Creatine uptake promotes dendritic cell activation and enhances antitumor immunity

**DOI:** 10.1016/j.isci.2026.115436

**Published:** 2026-03-21

**Authors:** Elliot Kang, James Elsten-Brown, Yu-Chen Wang, Ashley Lam, Elise Sanchez, Renee Wen, Tiffany Wang, Jennifer Chiang, Quentin Scarborough, Yan-Ruide Li, Yichen Zhu, Jie Huang, Matthew Williams, Sarah Eckl, Bo Li, Lili Yang

**Affiliations:** 1 Department of Microbiology, Immunology and Molecular Genetics, University of California, Los Angeles, Los Angeles, CA 90095, USA; 2 Department of Cardiovascular Surgery, First Affiliated Hospital of Anhui Medical University, and School of Basic Medical Sciences, Anhui Medical University, Hefei, Anhui 230032, China; 3 Department of Bioengineering, University of California, Los Angeles, Los Angeles, CA 90095, USA; 4 Eli and Edythe Broad Center of Regenerative Medicine and Stem Cell Research, University of California, Los Angeles, Los Angeles, CA 90095, USA; 5 Jonsson Comprehensive Cancer Center, the David Geffen School of Medicine, University of California, Los Angeles, Los Angeles, CA 90095, USA; 6 Goodman-Luskin Microbiome Center, University of California, Los Angeles, Los Angeles, CA 90095, USA; 7 Molecular Biology Institute, University of California, Los Angeles, Los Angeles, CA 90095, USA; 8 Parker Institute for Cancer Immunotherapy, University of California, Los Angeles, Los Angeles, CA 90095, USA

**Keywords:** immunology, immunological methods, cancer

## Abstract

Dendritic cells (DCs) are central regulators of antitumor T cell immunity and are highly sensitive to metabolic cues. However, the therapeutic potential of targeting DC metabolism remains underexplored. Here, we report upregulation of the creatine transporter (*CrT*; *Slc6a8)* in intratumoral DCs, which facilitates the cellular uptake of creatine, an energy-storage metabolite. DCs from *CrT* knockout mice exhibited impaired activation and reduced ability to elicit antigen-specific CD8 T cell responses. Conversely, creatine supplementation enhanced mouse DC activation *in vitro* and *in vivo*, and suppressed tumor growth in a syngeneic melanoma model. Notably, creatine uptake similarly boosted the activation and immunostimulatory function of human monocyte-derived DCs. Mechanistically, CrT promotes DC activation by preserving intracellular ATP levels and enhancing energy-dependent inflammatory signaling pathways. Together, these findings uncover a previously unrecognized role for creatine metabolism in regulating DC function and support the use of creatine supplementation as a strategy to augment DC-based cancer immunotherapy.

## Highlights

• Dendritic cells upregulate creatine transporter (CrT) upon stimulation
• Creatine supplementation enhances dendritic cell function by modulating ATP balance
• Creatine treatment induces favorable intratumoral dendritic cell phenotypes

## Graphical abstract

**Figure:**
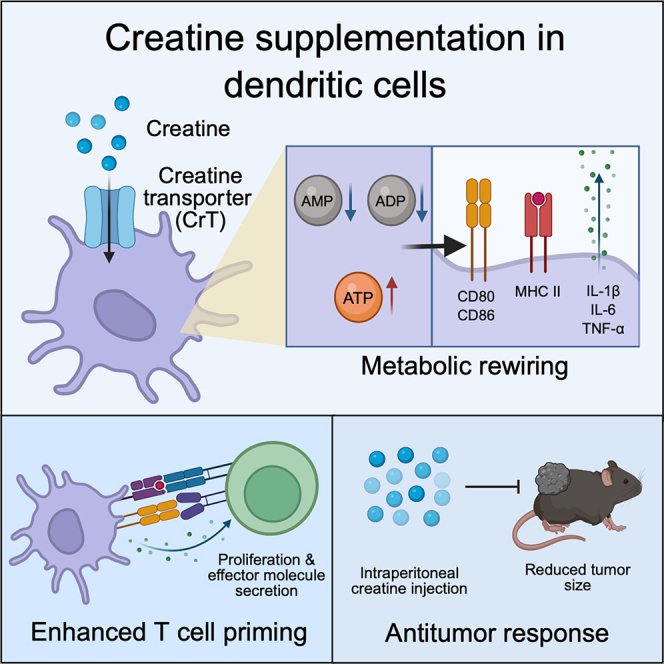

## Introduction

Dendritic cells (DCs) are a diverse group of specialized myeloid-derived antigen-presenting cells (APCs) that play a critical role in regulating antigen-specific immunity and tolerance.^1^^,^^2^ These immune cells bridge the innate and adaptive immune systems, facilitating the elimination of tumor cells. Upon activation via pattern recognition receptors (PRRs), DCs capture tumor antigens released into the tumor microenvironment (TME) and present these antigens via major histocompatibility complex (MHC) molecules to naive T cells in lymphoid tissues, activating tumor-specific T cell responses.^3^^,^^4^^,^^5^^,^^6^ Moreover, DCs produce cytokines (e.g., TNF- α, IL-6, IL-12, IFNs, CXCL9/10, etc.), which activate innate immune cells (e.g., natural killer [NK] cells, macrophages, and mast cells) and adaptive cytotoxic CD8 and “helper” CD4 T cells to orchestrate a comprehensive antitumor immune response.^6^^,^^7^^,^^8^ Consequently, DCs are key players in the TME and represent promising targets for cancer immunotherapy.^3^

Previous studies have associated the presence of intratumoral DCs with activated effector T cell phenotypes in the TME and favorable prognosis among patients with cancer.^6^^,^^9^^,^^10^^,^^11^^,^^12^^,^^13^ A lack of DCs in the TME or dysregulated DC function correlates with poorly immunogenic tumor types.^3^^,^^14^^,^^15^^,^^16^ Identification of new mechanisms altering DC effector functions are of significant interest and could offer new therapeutic strategies to enhance T cell responses against tumors, particularly among patients with poorly immunogenic and non-inflamed tumor types.^17^

Energy metabolism plays a critical role in the activation and function of DCs.^18^^,^^19^ DCs leverage multiple metabolic pathways, including oxidative phosphorylation, glycolysis, fatty acid oxidation (FAO), and glutaminolysis, to generate ATP, which support their homeostatic maintenance and effector functions.^18^^,^^19^^,^^20^^,^^21^ In the TME, metabolic reprogramming is a crucial hallmark.^22^ A disrupted metabolic balance due to competition with tumor cells for nutrients often leads to impaired DC activity and reduced antitumor immune responses.^18^^,^^19^^,^^23^ Understanding how metabolism regulates intratumoral DCs opens new avenues for developing cancer therapies that target key metabolic regulators to restore DC function.

Creatine is a nitrogenous organic acid capable of storing high-energy phosphates and buffering intracellular ATP levels through a CK/PCr/Cr (creatine kinase/phosphocreatine/creatine) system.^24^ Creatine supplements have been broadly used by bodybuilders and athletes to gain muscle mass and to improve performance.^24^^,^^25^^,^^26^ However, the function of creatine/creatine transporter (CrT; SLC6A8) in the immune system, especially in cancer immunity, is largely unknown. Our group and others have identified creatine as an important metabolic regulator intrinsically enhancing the antitumor CD8 T cell response^27^ and polarizing intratumoral macrophages into the proinflammatory M1 phenotype.^28^ These findings, highlighting creatine’s immunomodulatory role within the TME, directed our focus toward DCs, which are central components of the TME. In this study, we investigated the role of CrT in regulating DC activation and evaluated the potential of creatine supplementation to enhance DC-mediated antitumor immunity using genetic and pharmacological approaches, multi-omics analyses, and preclinical syngeneic mouse tumor models.

## Results

### Creatine uptake enhances dendritic cell survival and activation

**Figure 1:**
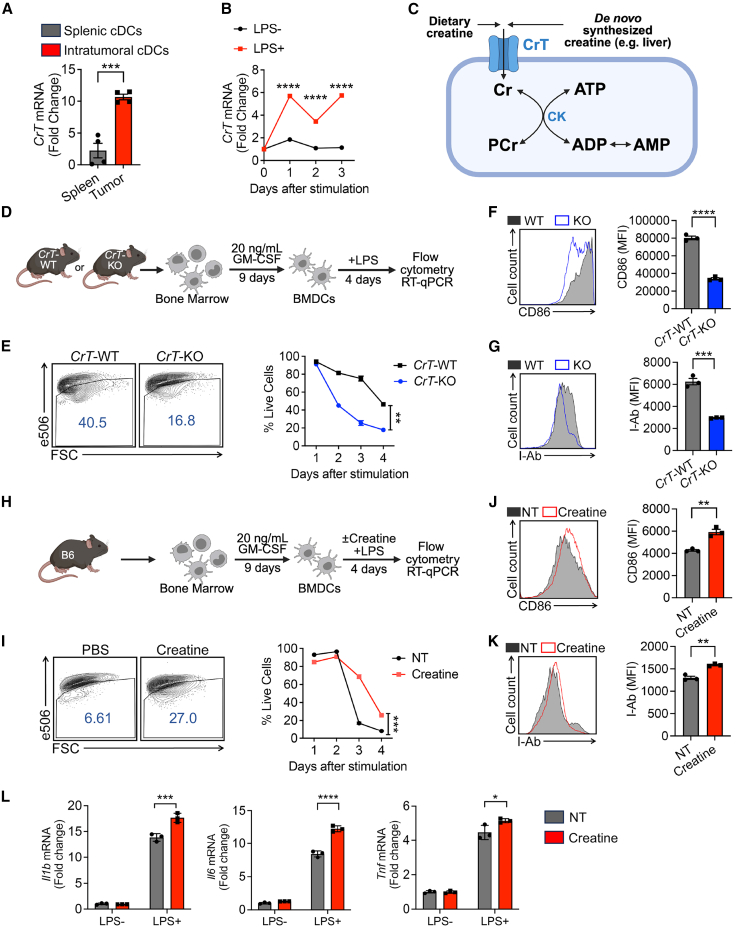
Creatine uptake enhances dendritic cell survival and activation (A) Quantitative reverse-transcription PCR (RT-qPCR) analysis of *CrT* in intratumoral cDCs (gated as CD45.2^+^CD11c^+^I-Ab^hi^) isolated from day 14 B16-OVA tumors grown in wild-type B6 mice (*n* = 4). Splenic cDCs were included as a control (*n* = 4). (B) RT-qPCR analysis of *CrT* mRNA expression in bone marrow-derived dendritic cells (BMDCs) treated with 100 ng/mL LPS (*n* = 3). Unstimulated cells were included as a control (*n* = 3). (C) Diagram showing creatine uptake and creatine-mediated bioenergy buffering in cells with high-energy demand. Cr, creatine; PCr, phosphocreatine; CrT, creatine transporter; CK, creatine kinase. (D–G) BMDC activation in the absence of CrT. (D) Experimental design. (E–G) FACS analyses of viability at day 4 (E) and CD86 (F), and I-Ab (G) levels at day 3 in *CrT*-WT and *CrT*-KO BMDCs treated with 100 ng/mL LPS (*n* = 3). MFI, median fluorescence intensity. (H–L) BMDC activation under creatine treatment. (H) Experimental design. (I–K) FACS analyses of viability at day 4 (I) and CD86 (J), and I-Ab (K) levels at day 3 in BMDCs treated with 0.5 mM creatine and 100 ng/mL LPS (*n* = 3). (L) RT-qPCR analyses of *Il1b*, *Il6*, and *Tnf* expression in BMDCs treated with 0.5 mM creatine and 10 ng/mL LPS at 24 h (*n* = 3). Representative of one (A), two (B–G), and three (H–L) experiments. Data are presented as the mean ± SEM. ^∗^*p* < 0.05, ^∗∗^*p* < 0.01, ^∗∗∗^*p* < 0.001, and ^∗∗∗∗^*p* < 0.0001 by Student’s *t* test (A, E–G, and I–K) adjusted for multiple comparisons (B and L). See also Figure S1.

To identify metabolic regulators of DC function, we isolated murine CD11c^+^I-Ab^hi^ intratumoral and splenic conventional DCs (cDCs). We then assessed the expression of nutrient-related genes using quantitative RT-PCR (qPCR). Splenic cDCs were included as controls. Interestingly, we detected a marked upregulation of the creatine transporter (*CrT*) in intratumoral cDCs compared to controls (Figure 1A). *CrT* was also highly upregulated in cultured bone marrow-derived DCs (BMDCs) stimulated *in vitro* with lipopolysaccharide (LPS) compared to controls (Figure 1B). CrT is an X-linked, sodium- and potassium-dependent transporter responsible for intracellular creatine uptake.^29^^,^^30^ Once imported into cells, creatine participates in the CK/PCr/Cr system (Figure 1C),^25^^,^^31^ which has been implicated in maintaining ATP homeostasis in cells with high metabolic demands.^27^^,^^32^

To address whether the CrT/creatine system acts as an autonomous factor directly regulating DC activity, we cultured BMDCs from wild-type (*CrT*-WT) or CrT knockout (*CrT*-KO) mice *in vitro* and compared their survival and activation upon LPS stimulation (Figure 1D). We found *CrT*-KO DCs showed a dramatic decrease in cell survival (Figure 1E) and activation, characterized by downregulated expression of surface activation markers (i.e., CD86 and I-Ab; Figures 1F and 1G). Production of proinflammatory cytokines (i.e., TNF-α and IL-6) was also significantly decreased (Figure S1).

To investigate whether creatine uptake directly contributed to the hyperresponsiveness of DCs, we cultured BMDCs from *CrT*-WT mice and stimulated them with LPS in the presence or absence of creatine (Figure 1H). Creatine supplementation resulted in a significant enhancement in DC survival (Figure 1I) and expression of surface activation markers (i.e., CD86 and I-Ab, Figures 1J and 1K) and proinflammatory cytokines (i.e., IL-1β, IL-6, and TNF-α; Figure 1L).

Collectively, these *in vitro* studies indicate that mouse DCs, after LPS stimulation, increase their capacity to uptake creatine that enhances their survival and activation.

### Creatine uptake deficiency impairs dendritic cell-mediated T cell antigen responses

**Figure 2:**
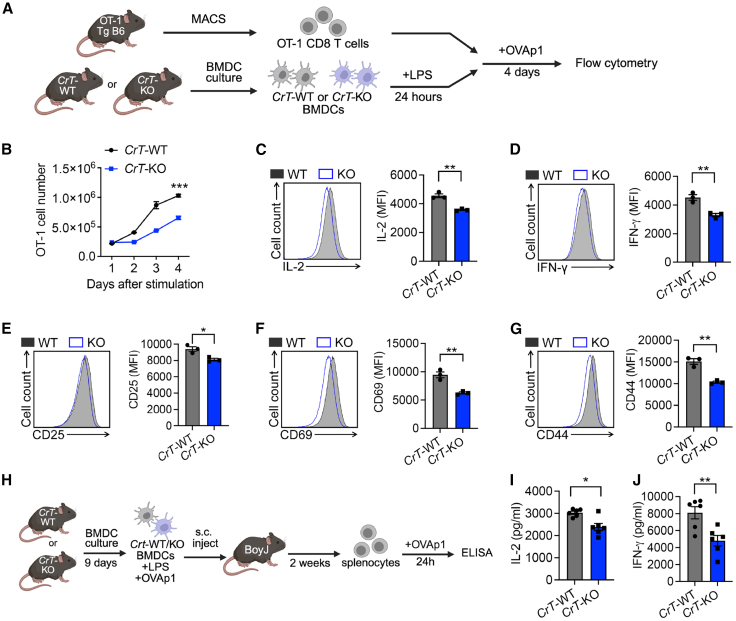
Creatine uptake deficiency impairs dendritic cell-mediated T cell antigen responses (A–G) T cell activation by *CrT*-WT or *CrT*-KO BMDCs. (A) Experimental design. *CrT*-WT or *CrT*-KO LPS-activated (100 ng/mL) BMDCs loaded with OVAp1 (0.1 μg/mL) were cocultured with CD8 T cells isolated from OT1-Tg mice. MACS, magnetic-activated cell sorting. (B–D) FACS analysis of OT-1 T cell counts (B); IL-2 (C) and IFN-γ production (D); and CD25 (E), CD69 (F), and CD44 (G) expression in CD8^+^ cells at day 4 (*n* = 3). (H–J) DC adoptive transfer experiment. (H) Experimental design. *CrT*-WT and *CrT*-KO BMDCs were stimulated with 100 ng/mL LPS for 24 h and loaded with OVAp1 for 1 h prior to subcutaneous injection of 1 × 10^6^ cells into BoyJ mice. At day 14, 1 × 10^6^ total splenocytes were seeded and restimulated with OVAp1 to measure anti-OVAp1 responses. (I and J) ELISA measurement of total secreted IL-2 (I) and IFN-γ (J) by restimulated splenocytes (*n* = 6). Representative of one (H–J) and two (A–G) experiments. Data are presented as the mean ± SEM. ^∗^*p* < 0.05, ^∗∗^*p* < 0.01, and ^∗∗∗^*p* < 0.001 by Student’s *t* test. See also Figure S2.

To assess whether creatine uptake deficiency could impact DC induction of T cell antigen responses, we performed an *in vitro* DC/T cell co-culture assay. OT1 T cells were isolated from OT1 transgenic (OT1-Tg) mice and co-cultured at a 10:1 ratio with LPS-stimulated BMDCs from either *CrT*-WT or *CrT*-KO mice in the presence of the cognate OVA peptide (Figure 2A). After 4 days of co-culture, OT1 T cells stimulated by *CrT*-KO BMDCs exhibited significantly reduced proliferation and viability compared to those co-cultured with *CrT*-WT BMDCs (Figures 2B and S2. In addition, OT1 T cells in the *CrT*-KO BMDC co-culture produced lower levels of effector cytokines (i.e., IL-2 and IFN-γ; Figure 2C) and expressed reduced levels of canonical activation markers (i.e., CD25, CD69, and CD44; Figures 2E–2G). These findings indicate that DCs lacking creatine uptake exhibit a diminished capacity to prime antigen-specific T cell responses, presumably due to their impaired expression of costimulatory ligands and cytokines.

To validate whether the creatine/CrT system directly regulates DC function and thereby influences T cell antigen responses *in vivo*, we performed a BMDC adoptive transfer experiment. OVA peptide-loaded BMDCs grown from *CrT*-WT or *CrT*-KO mice were subcutaneously (s.c.) injected into BoyJ WT-recipient mice, and splenocytes from these mice were isolated two weeks later and restimulated with OVA peptide *ex vivo* for 24 h (Figure 2H). ELISA analyses of *ex vivo* culture medium showed the splenocytes from mice receiving *CrT*-KO BMDCs produced significantly lower levels of canonical T cell effector cytokines (i.e., IL-2 and IFN-γ; Figures 2I and 2J). This indicated an impaired recall immune response in mice with *CrT*-KO BMDC transfer, potentially mediated by the reduced DC viability and effector phenotype.

Collectively, these *in vitro* and *ex vivo* studies demonstrate that CrT acts as an autonomous factor positively regulating DC promotion of T cell antigen responses.

### Creatine uptake enhances dendritic cell activation by regulating ATP/energy buffering

**Figure 3:**
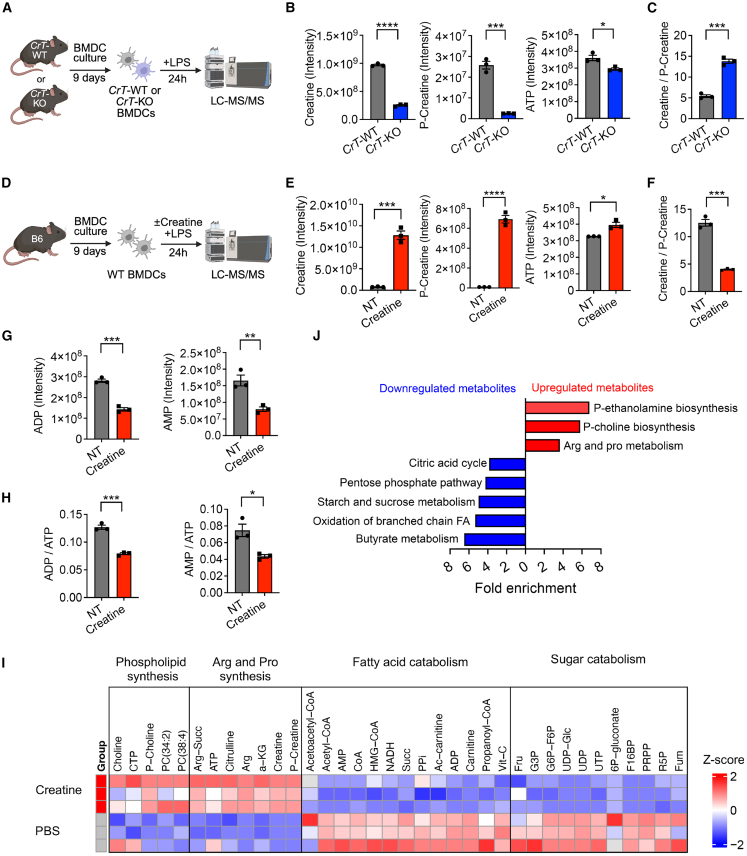
Creatine uptake enhances dendritic cell activation by regulating ATP/energy buffering (A–C) Liquid chromatography-tandem mass spectrometry (LC-MS/MS) metabolomics examination of *CrT*-WT and *CrT*-KO LPS-activated (100 ng/mL) BMDCs at 24 h. (A) Experimental design. (B) Creatine, phosphocreatine, and ATP levels of *CrT*-WT and *CrT*-KO BMDCs (*n* = 3). (C) Creatine/phosphocreatine ratio (*n* = 3). (D–J) LC-MS/MS metabolomics examination of creatine-treated LPS-activated BMDCs at 24 h. (D) Experimental design. (E) Creatine, phosphocreatine, and ATP levels of creatine-treated and control BMDCs (*n* = 3). (F) Creatine/phosphocreatine ratio (*n* = 3). (G and H) ADP and AMP levels (G) and ratios with ATP (H) (*n* = 3). (I and J) Enrichment analysis of metabolic pathways in creatine-treated BMDCs. (I) Heatmap showing key differential metabolites. Each row represents a biological replicate. (J) Bar plots showing the fold enrichment of indicated pathways in creatine-treated BMDCs. The experiment was performed once. Data are presented as the mean ± SEM. ^∗^*p* < 0.05, ^∗∗^*p* < 0.01, ^∗∗∗^*p* < 0.001, and ^∗∗∗∗^*p* < 0.0001 by Student’s *t* test.

Many cell types, including immune cells, are known to uptake creatine to buffer intracellular ATP levels and support cellular functions through the CK/PCr/Cr system.^27^^,^^28^^,^^33^ Therefore, we investigated whether activated DCs might utilize a similar mechanism to boost their energy metabolism. BMDCs from *CrT*-WT or *CrT*-KO mice were stimulated with LPS and analyzed using liquid chromatography-tandem mass spectrometry (LC-MS/MS)-based metabolomics (Figure 3A). Compared to *CrT*-WT BMDCs, *CrT*-KO BMDCs exhibited markedly lower levels of intracellular creatine and phosphocreatine, along with significantly reduced ATP levels (Figure 3B). Furthermore, the creatine-to-phosphocreatine ratio was substantially higher in *CrT*-KO BMDCs (Figure 3C), suggesting a diminished capacity for energy buffering.

To assess whether creatine supplementation enhances energy buffering in *CrT*-WT BMDCs, we cultured BMDCs from *CrT*-WT mice and stimulated them with LPS in the presence or absence of creatine (Figure 3D). Creatine-treated BMDCs showed significantly increased intracellular levels of creatine, phosphocreatine, and ATP (Figure 3E). Notably, the creatine-to-phosphocreatine ratio was markedly reduced in these cells (Figure 3F). Consistent with elevated ATP levels, creatine-treated BMDCs exhibited decreased intracellular ADP and AMP levels (Figure 3G), along with significantly lower AMP-to-ATP and ADP-to-ATP ratios (Figure 3H), indicating improved energy homeostasis and buffering capacity compared to vehicle-treated controls.

To further investigate the metabolic effects of creatine supplementation, we performed pathway analysis on metabolites specifically upregulated or downregulated by creatine treatment. Metabolites enriched in creatine-supplemented BMDCs were associated with phospholipid biosynthesis and arginine and proline metabolism (Figures 3I and 3J). In contrast, pathways related to the citric acid cycle, fatty acid metabolism, and sugar catabolism showed markedly lower enrichment (Figures 3I and 3J), suggesting that creatine supplementation may alleviate metabolic stress and promote biosynthesis in activated DCs.

Together, these *in vitro* metabolomic profiling studies suggest that creatine uptake may play an important role in regulating bioenergy homeostasis of activated DCs.

### Creatine uptake enhances dendritic cell activation by directly regulating TLR signaling

**Figure 4:**
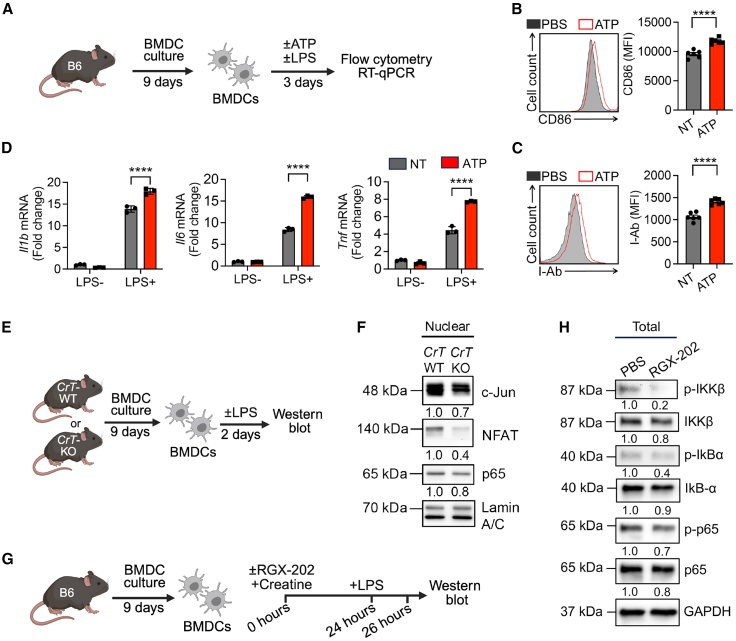
Creatine uptake enhances dendritic cell activation by directly regulating TLR signaling (A–D) BMDC activation under ATP treatment. (A) Experimental design. (B and C) FACS analysis of CD86 (B) and I-Ab (C) levels in ATP-treated BMDCs activated with 100 ng/mL LPS at day 3 (*n* = 6). (D) RT-qPCR analysis of *Il1b*, *Il6*, and *Tnf* expression in ATP-treated BMDCs activated with 10 ng/mL LPS at 24 h (*n* = 3). (E and F) Nuclear transcription factor analysis of *CrT*-KO BMDCs. (E) Experimental design. (F) Western blot analysis of DC activation transcription factors in *CrT*-WT and *CrT*-KO BMDCs. (G and H) NF-κB signaling cascade inhibition experiment. (G) Experimental design. (H) Western blot analysis of phosphorylated NF-κB signaling molecules in control and creatine transporter inhibitor (RGX-202)-treated BMDCs after 2 h of LPS stimulation (100 ng/mL). Representative of two (E–H) and three (A–D) experiments. Data are presented as the mean ± SEM. ^∗∗∗∗^*p* < 0.0001 by Student’s *t* test (B and C) adjusted for multiple comparisons (D). See also Figures S3 and S4.

To study the direct regulation of DCs by ATP, we supplemented exogenous ATP into cultures of *CrT*-WT BMDCs upon LPS stimulation (Figure 4A). Extracellular ATP supplementation further enhanced the activation of *CrT*-WT BMDCs, as evidenced by upregulated expression of DC surface activation markers (i.e., CD86 and I-Ab, Figures 4B and 4C) and enhanced gene expression of proinflammatory cytokines (i.e., IL-1β, IL-6, and TNF-α, Figure 4D). Furthermore, previous studies have demonstrated that intracellular delivery of ATP enhanced BMDC activation, underscoring its role in DC function.^34^ ATP supplies bioenergy and high-energy phosphate groups for immune signaling pathways.^35^ We therefore hypothesized that the CK/PCr/Cr ATP-buffering system might regulate BMDC activity through modulating inflammatory PRR signaling pathways.

To test this, we compared the major Toll-like receptor (TLR) downstream signaling pathways between *CrT*-WT and *CrT*-KO BMDCs (Figure 4E). We found that creatine uptake deficiency impeded activation and nuclear translocation of several TLR-associated downstream transcription factors, including nuclear factor of activated T cells (NFAT), c-Jun (Jun protooncogene, AP-1 transcription factor subunit), and p65 (nuclear factor κB [NF-κB] subunit), which may account for the hypoactivation of *CrT*-KO BMDCs (Figure 4F). Consistently, creatine supplementation significantly enhanced p65 nuclear translocation in *CrT*-WT BMDCs, and a similar enhancement was observed upon ATP supplementation (Figures S3A and S3B).

Notably, among the transcription factors examined, NF-κB signaling—particularly the p65 subunit—appeared most responsive to fluctuations in intracellular ATP. Both creatine and ATP supplementation enhanced p65 activity, whereas CrT deficiency reduced it (Figures 4F and S3B), suggesting that NF-κB may act as an ATP-sensitive signaling node during DC activation. To further assess the role of NF-κB in mediating creatine-driven BMDC activation, we treated *CrT*-WT BMDCs with the small-molecule creatine transporter inhibitor RGX-202. Alongside impairing BMDC activation (Figures S2C–S2E), RGX-202 treatment markedly deactivated NF-κB signaling, as shown by a significant reduction in phosphorylation of IKKβ, IκBα, and p65 in the cytoplasm (Figure 4H).

As the previous studies were conducted exclusively using LPS, a TLR4 agonist, we confirmed a similar enhanced increase in activation markers (e.g., CD80 and CD86) upon TLR3 stimulation with poly (I:C) in both creatine- and ATP-treated DCs (Figure S4). Thus, the impact of creatine-induced increase in ATP availability is not restricted to TLR4 signaling and potentially has broad effects across multiple stimulation pathways.

Collectively, these results support an intriguing working model in which activated DCs rely on a potent creatine-mediated ATP/energy buffering system to sustain PRR signaling, thereby powering DC functions.

### Creatine supplementation enhances mouse intratumoral cDC activation

**Figure 5:**
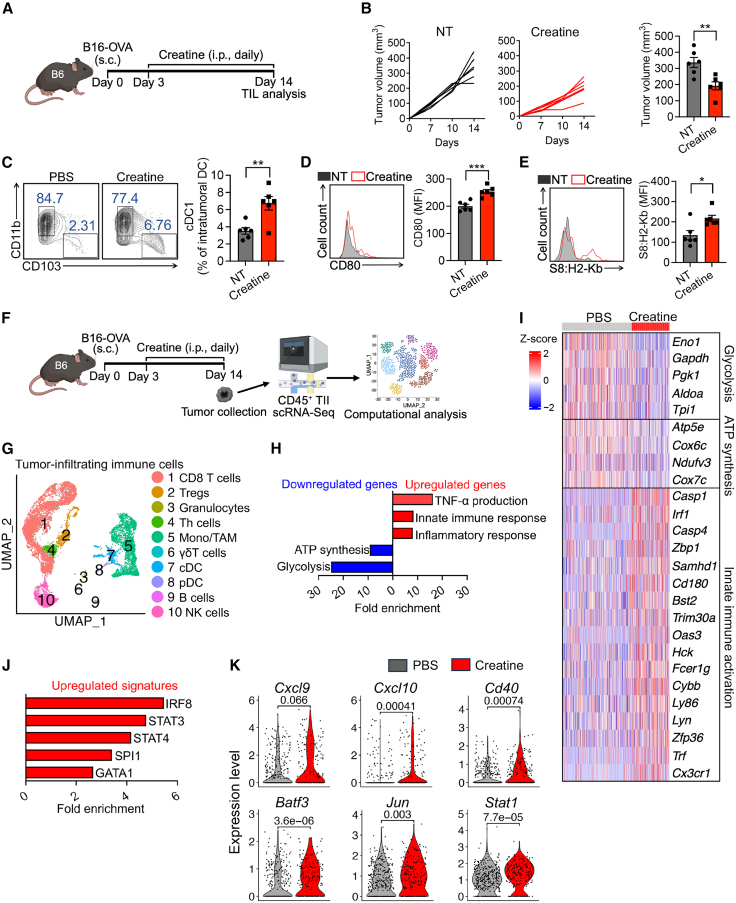
Creatine enhances intratumoral mouse cDC activation (A–E) Creatine treatment in tumor therapy experiments. (A) Experimental design. Mice were intraperitoneally injected with 10.5 mg of creatine daily beginning day 3 after tumor inoculation. (B) Tumor growth (*n* = 6). (C) FACS analysis of intratumoral cDC1 and cDC2 proportions (*n* = 6). (D and E) FACS analysis of CD80 (D) and OVAp1 presented on H2-K^b^ (E) levels in cDC1 subsets (gated as CD45.2^+^CD11c^+^I-A^bhi^CD11b^lo^CD103^+^) (*n* = 6). (F–K) Single-cell RNA sequencing analysis of creatine-treated mice. (F) Experimental design. (G) Total CD45^+^ TIIs combined from all samples. Combined uniform manifold approximation and projection (UMAP) plot is presented, showing the formation of ten major cell clusters. Each dot represents a single cell and is colored according to its cell cluster assignment. (H) Bar plot showing the fold enrichment of indicated pathways in genes upregulated or downregulated in creatine-treated cDCs compared to control cDCs. (I) Heatmap showing the expression of representative genes in each treatment group. Each column represents an individual cell. (J) Transcription factor target enrichment analysis. Bar plot showing the fold enrichment of indicated transcription factor target genes in creatine-treated cDCs. (K) Violin plots of key cDC activation genes. Each dot represents an individual cell. *p* values from the Wilcoxon test are shown. Representative of three experiments (A–F). Data are presented as the mean ± SEM. ^∗^*p* < 0.05, ^∗∗^*p* < 0.01, and ^∗∗∗^*p* < 0.001, by Student’s *t* test (A–F). Single-cell RNA sequencing was performed once, and cells isolated from five mice of each experimental group were combined for analysis (G–K). The *p* values of violin plots were determined by the Wilcoxon test (K). *p* < 0.05 was considered significant. See also Figures S5–S7.

The proposed “creatine-uptake/energy-buffering” model presents a metabolic strategy to enhance intratumoral cDC-mediated antitumor immunity through creatine supplementation. To evaluate this new concept *in vivo*, we supplemented creatine to experimental *CrT*-WT mice in the B16-OVA melanoma model (Figure 5A). Notably, creatine treatment significantly reduced tumor growth (Figure 5B). Analysis of day 14 B16-OVA tumors revealed increased abundance of type 1 cDCs (cDC1s), the most potent tumor antigen-presenting cDCs,^36^ in creatine-treated mice (Figure 5C), accompanied by enhanced activation, as evidenced by increased expression of CD80 (Figure 1D). Further analysis revealed a higher abundance of OVA tumor antigen-specific cDC1s in creatine-treated tumors (Figure 5E). Follow-up *in vitro* analyses utilizing bone marrow-derived cDC1s (BMDC1s) showed higher CD80, CD86, and I-Ab expression and increased TNF-α and IL-6 production in response to poly (I:C) stimulation upon creatine supplementation (Figure S5).

To assess how creatine supplementation affects intratumoral cDCs at a transcriptional level, we isolated CD45^+^ tumor-infiltrating immune cells (TIIs) from B6 mice bearing B16-OVA tumors treated with or without creatine and conducted a single-cell RNA sequencing (scRNA-seq) study (Figure 5F). Uniform manifold approximation and projection (UMAP) analysis of total combined TIIs showed the formation of ten cell clusters, including CD8 T cells, CD4 regulatory T cells (Tregs), granulocytes, CD4 helper T cells (Th cells), monocytes/tumor-associated macrophages (Mono/TAM), γδ T cells, B cells, NK cells, cDCs, and plasmacytoid DCs (pDCs) (Figures 5G and S6). We further performed pathway analysis for genes specifically upregulated or downregulated by creatine supplementation in the cDC cluster. Creatine supplementation-enriched pathways were related to inflammatory pathways, such as TNF-α production, innate immune response, and inflammatory response. On the other hand, downregulated pathways included metabolic processes, such as ATP synthesis and glycolysis (Figures 5H and 5I).

Additionally, transcription factor target enrichment analysis revealed gene expression upregulation of the key transcription factor signatures (i.e., IRF8, STAT3, STAT4, SPl1, and GATA1) for innate immune responses in the creatine-treated cDCs (Figure 5J). Consistently, single-gene expression analysis also showed an overall enhanced expression of genes associated with DC activation (i.e., *Cxcl9*, *Cxcl10*, *Cd40*, *Batf3*, *Jun*, and *Stat1*; Figure 5K) in tumor-infiltrating cDCs isolated from mice with creatine supplementation.

To identify the effects of enhanced cDC inflammatory phenotypes on T cells, we examined the CD8 T cell cluster in our dataset. Notably, pathways relevant to T cell activation, effector functions, and communication with DCs were enriched in CD8 T cells isolated from creatine-treated mice, demonstrating a potential direct explanation for the observed decrease in tumor burden (Figure S7).

Together, these *in vivo* gene profiling studies suggest that creatine uptake plays a critical role in regulating cDC activation and enhancing cDC-mediated antitumor immunity.

### Creatine uptake enhances human MoDC activation and function

**Figure 6:**
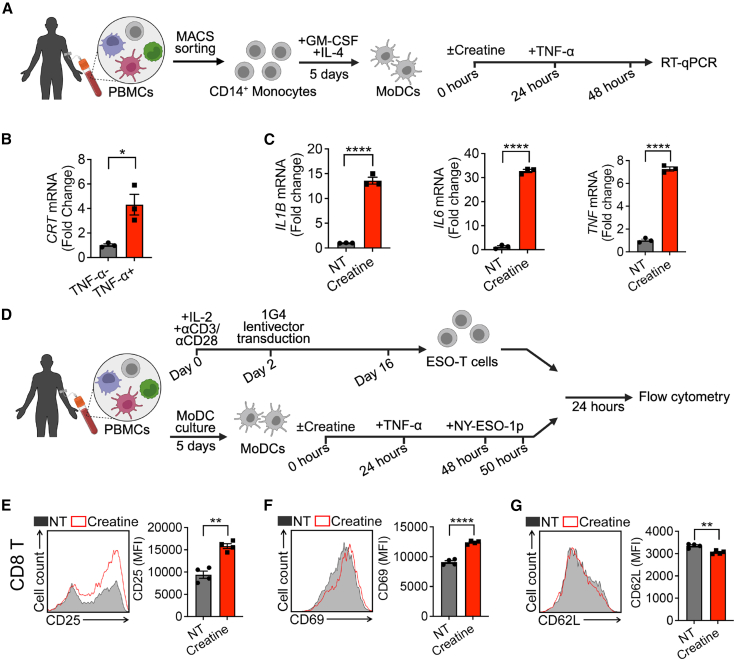
Creatine enhances human MoDC activation and function (A–C) Human monocyte-derived dendritic cell (MoDC) activation under creatine treatment. (A) Experimental design. (B) RT-qPCR analysis of *CRT* in MoDCs with and without TNF-⍺ (50 ng/mL) stimulation (*n* = 3). (C) RT-qPCR analysis of *IL1B*, *IL6*, and *TNF* expression in MoDCs treated with 50 mM creatine and 50 ng/mL TNF-⍺ (*n* = 3). (D–G) T cell activation by creatine-treated MoDCs. (D) Experimental design. Control or creatine-treated MoDCs loaded with NY-ESO-1 peptide (5 μM) were cocultured with T cells engineered to express ESO-TCR. (E–G) FACS analysis of CD25 (E), CD69 (F), and CD62L (G) expression in CD8^+^ ESO-T cells (*n* = 5). Representative of two experiments. Data are presented as the mean ± SEM. ^∗^*p* < 0.05, ^∗∗^*p* < 0.01, ^∗∗∗^*p* < 0.001, and ^∗∗∗∗^*p* < 0.0001 by Student’s *t* test. See also Figure S8.

To assess the translational potential of creatine supplementation as an immunomodulatory therapy, we studied creatine regulation of human DC activation. Human monocyte-derived DCs (MoDCs) were cultured from peripheral blood mononuclear cells (PBMCs) from healthy donors *in vitro* and stimulated with human TNF-α in the presence or absence of creatine (Figures 6A and S8A). In response to TNF-α stimulation, human MoDCs showed a marked increase in the gene expression of *CRT* (Figure 6B), resembling our findings in mouse BMDCs. Of note, creatine-treated human MoDCs showed enhanced activation, characterized by upregulated expression of proinflammatory cytokines (i.e., *IL1B*, *IL6*, and *TNF*; Figure 6C).

Next, we investigated whether creatine-induced activation of human DCs could enhance T cell responses to tumor antigens using a human MoDC-T cell co-culture system. NY-ESO-1, a well-recognized tumor antigen commonly expressed in a large variety of human cancers,^37^ was chosen as the model tumor antigen. NY-ESO-1-specific human T cells were generated by transducing healthy donor PBMCs with a lentivector encoding an NY-ESO-1-specific TCR^38^ (denoted as ESO-T cells; Figure S8B). MoDCs from HLA-A2^+^ donors were treated stimulated with TNF-α in presence or absence of creatine, and loaded with NY-ESO-1 peptide, followed by co-culture with ESO-T cells (Figures 6D and S8C). CD8 ESO-T cells from ESO-T co-cultured with creatine-treated human MoDCs exhibited enhanced activation status, evidenced by significantly upregulated expression of CD25 and CD69 and downregulated expression of CD62L (Figures 6E–6G). Consistent results were also obtained in CD4 ESO-T cell population from the co-culture (Figures S8D–S8F).

These *in vitro* human data support creatine as a positive regulator of human DC function, illustrating its potential as a promoter of tumor immunogenicity in next-generation cancer immunotherapy strategies.

## Discussion

**Figure 7:**
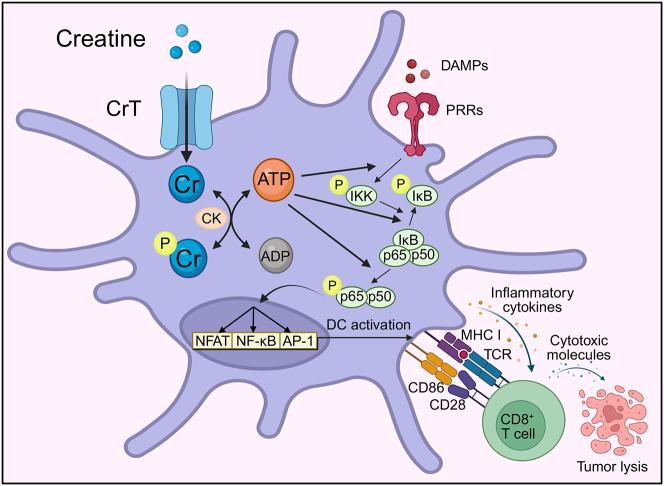
Creatine as a positive regulator of intratumoral DC activation and function Schematic showing regulation of intratumoral DC activation and antitumor immunity with creatine (Cr). In this model, creatine transporter (CrT) is highly expressed in intratumoral DCs, allowing high levels of creatine transport into the cytoplasm. Creatine is enzymatically converted to phosphocreatine (PCr) by creatine kinase (CK), serving as a cellular energy reserve. ATP regenerated from ADP via CK is utilized to enhance inflammatory signaling from innate pattern recognition receptors (PRRs) binding to damage-associated molecular patterns (DAMPs) by facilitating phosphorylation of signaling pathways. Activation of inflammatory transcription factors induces expression of key T cell-activating costimulatory molecules, leading to antigen-specific CD8 T cell activation and antitumor immunity.

Immune cells, upon activation, require substantial energy to support biosynthetic pathways, intracellular signaling, and dynamic processes such as proliferation, differentiation, and migration.^39^^,^^40^ Recently, the creatine-mediated ATP/energy buffering system has emerged as a critical metabolic pathway in tumor immunology.^31^ Our group and others have demonstrated that creatine uptake directly enhances antitumor CD8 T cell responses and promotes proinflammatory polarization of intratumoral macrophages.^27^^,^^28^ Here, we uncover a previously unrecognized role for CrT in regulating DC-mediated antitumor immunity and propose a “creatine-uptake/energy-buffering” model of DC activation. Following PRR stimulation by damage-associated molecular patterns (DAMPs), intratumoral DCs upregulate CrT, facilitating the import of extracellular creatine (Figures 1 and 6). The imported creatine functions as an energy reservoir, buffering intracellular ATP derived from glycolysis and the tricarboxylic acid cycle (Figure 3).^18^ This bioenergetic support sustains ATP-dependent inflammatory signaling pathways and enhances DC capacity to prime CD8 T cell responses. These findings offer fundamental insights into the metabolic regulation of DC function and highlight an important axis driving antitumor immunity (Figure 7).

5′ adenosine monophosphate-activated protein kinase (AMPK) is an energy sensor that detects shifts in the AMP:ATP ratio and maintains energy homeostasis under conditions of energetic stress.^41^ In DCs, activation of the AMPK pathway in response to elevated AMP:ATP ratios promotes catabolic processes and suppresses lipid biosynthesis, thereby favoring a metabolically quiescent, anti-inflammatory state.^42^ In contrast, DC activation is an energy-intensive process that requires a metabolic shift toward anabolic pathways to support biosynthesis and immunostimulatory functions.^20^^,^^21^^,^^43^^,^^44^ In this study, creatine uptake led to a low AMP:ATP ratio in DCs (Figure 3), indicating a high-energy intracellular state. Additionally, our *in vitro* and *in vivo* data showed creatine-induced DC activation was accompanied by suppression of catabolic pathways, including glycolysis and FAO, and upregulation of phospholipid synthesis, a hallmark of energy-demanding anabolic reprogramming (Figures 3 and 5).^44^ These findings highlight the creatine-mediated ATP/energy buffering system as a key regulator of DC metabolic reprogramming within the TME, likely through modulation of AMPK signaling to promote a high-energy, immunostimulatory phenotype.

Recent studies have demonstrated the crucial role of cDCs in orchestrating antitumor immune responses.^1^^,^^3^^,^^8^ In many human cancers, higher intratumoral cDC1 abundance has been specifically associated with improved clinical response to immune checkpoint blockade (ICB) therapy.^4^^,^^45^^,^^46^ Our study showed that creatine supplementation significantly suppressed tumor growth in the mouse syngeneic B16-OVA melanoma model and increased the abundance and activation of intratumoral cDC1s (Figure 5). Although the increased activation of cDC1s was replicated in follow-up *in vitro* studies (Figure S5), the mechanism behind their overabundance remains unclear. Creatine uptake enhanced the capacity of human DCs to cross-present tumor antigens to CD8 T cells (Figure 6) and upregulated the expression of chemokines such as CXCL9 and CXCL10 (Figure 5), which facilitate the recruitment of tumoricidal CD8 T cells and NK cells into the TME.^8^^,^^47^^,^^48^^,^^49^ The current slate of approved ICB therapies manipulate antitumor immunity by directly targeting CD8 T cell exhaustion, limiting their efficacy in immunogenically cold, low-infiltration tumors.^46^ Our findings position creatine supplementation as a promising DC-targeted immunotherapy that may increase tumor immunogenicity, providing an economical, mechanistically nonredundant co-therapy strategy for overcoming ICB resistance.

Decreased serum creatine levels have been observed in cancer patients, particularly those with advanced disease who frequently experience cachexia, a severe wasting syndrome characterized by weight and muscle loss.^50^ Our group and others have shown that creatine supplementation can protect against cancer-associated cachexia in mouse models, preserving both body weight and muscle mass.^27^^,^^50^ Furthermore, creatine has been reported to attenuate doxorubicin-induced cardiotoxicity, a common side effect of chemotherapy.^51^ These multifaceted benefits of creatine supplementation illustrate its potential as a holistic, supportive strategy to enhance the efficacy and tolerability of a broad range of cancer treatments, including emerging immunotherapies, conventional chemotherapies, and radiation therapy.

As a ubiquitous energy metabolite, creatine may also directly influence cancer progression. Several studies have indicated that malignant cells can co-opt creatine metabolism to promote metastasis and disease progression.^52^^,^^53^ In particular, genetic or pharmacologic inhibition of the CrT or creatine kinase B has been associated with reduced tumor burden in specific models.^54^^,^^55^^,^^56^ However, these studies have not examined the impact of systemic creatine manipulation on antitumor immunity, nor have immunotherapy-focused studies thoroughly characterized the effects of creatine supplementation on tumor-intrinsic pathways. On the other hand, separate reports have demonstrated creatine’s ability to modulate CD8 T cell responses^27^ and macrophage polarization^28^ in a tumor context. More comprehensive understanding of creatine’s effects across distinct cellular components of the TME, including immune and malignant cells, is needed to fully define its therapeutic potential and context-specific outcomes.

In conclusion, we identify the creatine-mediated ATP/energy buffering system as a critical regulator of intratumoral DC activation and highlight the potential of creatine supplementation as a cancer immunotherapy. Our findings provide a mechanistic foundation for evaluating the clinical effects of creatine supplementation on antitumor immunity and exploring its potential synergy with existing immunotherapies. Indeed, a retrospective analysis of the United States National Health and Nutrition Examination Survey (NHANES) over 10 years revealed that higher dietary creatine intake was associated with a lower incidence of cancer.^57^ Given the well-documented safety profile of long-term creatine supplementation in humans, these strategies can be readily translated into clinical trials. Additionally, the enhanced functionality of creatine-treated human MoDCs, the most commonly used cells in DC vaccine platforms,^58^ suggests an additional opportunity to improve DC-based immunotherapies in future research.

### Limitations of the study

While our study demonstrates that creatine supplementation suppresses tumor growth and enhances intratumoral cDC-mediated antitumor immunity, it primarily relies on a single mouse syngeneic subcutaneous tumor model (B16-OVA). Future studies employing diverse, physiologically relevant models, such as orthotopic or patient-derived xenograft models, can further validate the therapeutic potential of creatine across tumor settings.

## Resource availability

### Lead contact

Further information and requests for resources and reagents should be directed to and will be fulfilled by the lead contact, Lili Yang (liliyang@ucla.edu).

### Materials availability

New viral constructs and cell lines generated in this study are available upon signing a material transfer agreement with UCLA.

### Data and code availability

All data associated with this study are present in the study or supplemental information. The scRNA-seq data generated in this study have been deposited at the Gene Expression Omnibus (GEO): GSE303921 and are publicly available as of the publication date. The metabolomics data have been deposited to MetaboLights repository^59^ with the study identifier MTBLS12797 and are publicly available as of the publication date.

Code and any additional information required to reanalyze the data reported in this study are available from the lead contact upon request.

## Acknowledgments

We thank Pin Wang (10.13039/100006034University of Southern California, CA, USA) for providing the B16-OVA cell line. We thank Gerald S. Lipshutz (University of California, Los Angeles, CA, USA) for providing the *CrT*-KO mice. We thank Timothy E. O’Sullivan (University of California, Los Angeles, CA, USA) for providing the BMDC1 culture protocol and reagents. We thank the UCLA animal facility for providing animal support; the UCLA Broad Stem Cell Research Centre (BSCRC) Flow Cytometry Core Facility for cell sorting support; the UCLA CFAR Virology Core Laboratory for providing human PBMCs; the UCLA Metabolomics Center for assisting with LC-MS/MS metabolomics; and the UCLA TCGB facility for assisting with scRNA-seq. This work was supported by a BSCRC-RHF Research Award from the Rose Hills Research Foundation (to L.Y.), a JCCC/BSCRC Ablon Scholars Award from UCLA (to L.Y.), a Magnolia Council Senior Investigator Grant Award from the Tower Cancer Research Foundation (to L.Y.), and a Parag Patel Research Support Fund (to L.Y.). J.E.B. is a predoctoral fellow supported by the Tower Cancer Research Foundation Fellowship. Y.-R.L. is a postdoctoral fellow supported by a UCLA MIMG M. John Pickett Post-Doctoral Fellow Award, a CIRM-BSCRC Postdoctoral Fellowship, a UCLA Sydney Finegold Postdoctoral Award, a UCLA Chancellor's Award for Postdoctoral Research, and a UCLA Goodman-Luskin Microbiome Center Collaborative Research Fellowship Award. Y.Z. is a predoctoral fellow supported by a Whitcome Pre-Doctoral Fellowship in Molecular Biology. M.W. and S.E. are undergraduate researchers supported by the Amgen Scholars Program.

## Author contributions

E.K., J.E.-B., B.L., and L.Y. designed and supervised the entire study. E.K. performed the experiments and analyzed the data, with assistance from J.E.-B. on *in vivo* studies; J.E.-B., A.L., E.S., R.W., J.C., Q.S., Y.Z., M.W., and S.E. on *in vitro* cell cultures; T.W. on western blot analysis; Y.-C.W. on cocultures, metabolomics, and scRNA-seq; and J.H. on human T cell studies. E.K., B.L., J.E.-B., Y.-R.L., and L.Y. wrote the manuscript.

## Declaration of interests

L.Y. is an inventor on patents relating to this study filed by UCLA. The authors declare no other competing financial interests.

## STAR★Methods

### Key resources table

**Table:** 

REAGENT or RESOURCE	SOURCE	IDENTIFIER
**Antibodies**		

Anti-mouse IFN-γ (ELISA, coating)	BD Biosciences	CAT#551216, RRID: AB_394094
Anti-mouse IFN-γ (ELISA, detection)	BD Biosciences	CAT#554410, RRID: AB_395374
Anti-mouse IL-2 (ELISA, coating)	BD Biosciences	CAT#554424; RRID: AB_395383
Anti-mouse IL-2 (ELISA, detection)	BD Biosciences	CAT#554426; RRID: AB_395384
Anti-mouse TNF-α (ELISA, coating)	Invitrogen	CAT#14-7325-81; RRID: AB_468481
Anti-mouse TNF-α (ELISA, detection)	Biolegend	CAT#506312; RRID: AB_315433
PE/Cyanine7 anti-mouse CD45.2 (Clone 104)	Biolegend	CAT#109830, RRID: AB_1186098
PerCP anti-mouse CD11b (Clone M1/70)	Biolegend	CAT#101230, RRID: AB_2129374
FITC anti-mouse CD11c (Clone N418)	Biolegend	CAT#117306, RRID: AB_313775
PE anti-mouse S8:H2-K^b^ (Clone 25-D1.16)	Biolegend	CAT#141604, RRID: AB_10895905
PE/Cyanine7 anti-mouse I-A^b^ (Clone AF6-120.1)	Biolegend	CAT#116420, RRID: AB_10575296
APC anti-mouse CD80 (Clone 16-10A1)	Biolegend	CAT#104714, RRID: AB_313135
APC/Cyanine7 anti-mouse CD86 (Clone GL-1)	Biolegend	CAT#105030, RRID: AB_2244452
APC/Cyanine7 anti-mouse TCRβ (Clone H57-597)	Biolegend	CAT#109220, RRID: AB_893624
FITC anti-mouse CD4 (Clone RM4-5)	Biolegend	CAT#100510, RRID: AB_312713
PerCP anti-mouse CD8a (Clone 53–6.7)	Biolegend	CAT#100732, RRID: AB_893423
APC anti-mouse CD69 (Clone H1.2F3)	Biolegend	CAT#104514, RRID: AB_492843
FITC anti-mouse CD25 (Clone PC61)	Biolegend	CAT#102006, RRID: AB_312855
Pacific Blue anti-mouse/human CD44 (Clone IM7)	Biolegend	CAT#103020, RRID: AB_493683
PE/Cyanine7 anti-mouse CD62L (Clone MEL-14)	Biolegend	CAT#104418, RRID: AB_313103
FITC anti-mouse IFN-γ (Clone XMG1.2)	Biolegend	CAT#505806, RRID: AB_315400
APC anti-mouse IL-2 (Clone JES6-5H4)	Thermo Fisher Scientific	CAT#17-7021-82, RRID: AB_469490
PE anti-mouse CD103 (Clone 2E7)	Biolegend	CAT#21406, RRID: AB_113398
Pacific Blue anti-mouse XCR1 (Clone ZET)	Biolegend	CAT#148241, RRID: AB_3674997
APC/Cyanine7 anti-mouse CD14 (Clone Sa14-2)	Biolegend	CAT#123312, RRID: AB_940575
FITC anti-mouse TNF-alpha (Clone MP6-XT22)	Biolegend	CAT#506304, RRID:AB_315425
PerCP/Cyanine5.5 anti-mouse IL-12/IL-23 p40 (Clone C15.6)	Biolegend	CAT#505212, RRID:AB_2566225
APC anti-mouse IL-6 (Clone MP5-20F3)	Biolegend	CAT#504508, RRID:AB_10694868
Pacific Blue anti-human CD45 (Clone HI30)	Biolegend	CAT#304022, RRID: AB_493655
PE anti-human CD3 (Clone OKT3)	Biolegend	CAT#317308, RRID: AB_571913
PerCP anti-human CD4 (Clone OKT4)	Biolegend	CAT#317432, RRID: AB_2028494
FITC anti-human CD8 (Clone SK1)	Biolegend	CAT#344704, RRID: AB_1877178
FITC anti-human CD69 (Clone FN50)	Biolegend	CAT#310904, RRID: AB_314839
FITC anti-human CD62L (Clone DREG-56)	Biolegend	CAT#304804, RRID: AB_314464
APC/Cyanine7 anti-human CD25 (Clone M-A251)	Biolegend	CAT#356122, RRID: AB_2562489
PE anti-human TCR Vβ13.1 (Clone H131)	Biolegend	CAT#362410, RRID: AB_2750159
Human Fc Receptor Blocking Solution (TrueStain FcX)	Biolegend	CAT#422302, RRID: AB_2818986
Mouse Fc Block (anti-mouse CD16/32)	BD Biosciences	CAT#553142, RRID: AB_394657
LEAF purified anti-human CD3 antibody (Clone HIT3a)	Biolegend	CAT#300314, RRID: AB_314050
LEAF purified anti-human CD28 antibody (Clone CD28.2)	Biolegend	CAT#302902, RRID: AB_314304
Anti-mouse NF-κB p65 (Clone D14E12)	Cell Signaling Technology	CAT#8242S, RRID: AB_10859369
Anti-mouse c-Jun (Clone 60A8)	Cell Signaling Technology	CAT#9165S, RRID: AB_2130165
Anti-mouse NFAT1	Cell Signaling Technology	CAT#4389S, RRID: AB_1950418
Anti-mouse phospho-IκBα (Clone 14D4)	Cell Signaling Technology	CAT#2859, RRID: AB_561111
Anti-mouse IκBα (Clone L35A5)	Cell Signaling Technology	CAT#4814, RRID: AB_390781
Anti-mouse phospho-IKKα/β (Clone 14D4)	Cell Signaling Technology	CAT#2697, RRID: AB_2079382
Anti-mouse IKKβ (Clone D30C6)	Cell Signaling Technology	CAT#8943, RRID: AB_11024092
Anti-mouse phospho-NF-κB p65 (Clone 93H1)	Cell Signaling Technology	CAT#3033, RRID: AB_331284
Anti-mouse IgG, HRP-linked antibody	Cell Signaling Technology	CAT#7076S, RRID: AB_330924
Anti-rabbit IgG, HRP-linked antibody	Cell Signaling Technology	CAT#7074S, RRID: AB_2099233
Anti-GAPDH (Clone 14C10)	Cell Signaling Technology	CAT#2118S, RRID: AB_561053
Anti-Lamin A/C (Clone 3A6-4C11)	Active Motif	CAT#39287, RRID: AB_2793218

**Bacterial and virus strains**		

Lenti/ESO-TCR	This paper	–

**Biological samples**		

Human peripheral blood mononuclear cells (PBMCs)	UCLA Center for AIDS Research (CFAR) Virology Core Laboratory	–

**Chemicals, peptides, and recombinant proteins**		

Lipopolysaccharides from Escherichia coli O111:B4	Sigma-Aldrich	CAT#L5293
Polyinosinic–polycytidylic acid sodium salt	Sigma-Aldrich	CAT#P1530
Streptavidin-HRP conjugate	Invitrogen	CAT#18410051
Mouse IFN-γ (ELISA, standard)	BioLegend	CAT#575309
Mouse IL-2 (ELISA, standard)	BioLegend	CAT#575409
Mouse TNF-α (ELISA, standard)	Thermo-Fisher Scientific	CAT#50-112-3494
Tetramethylbenzidine (TMB)	KPL	CAT#51200048
Creatine monohydrate	Sigma-Aldrich	CAT#C3630
Creatine transporter inhibitor RGX-202	MedChemExpress	CAT#HY-W0151828
RPMI1640 cell culture medium	Corning Cellgro	CAT#10-040-CV
DMEM cell culture medium	Corning Cellgro	CAT#10-013-CV
Fetal Bovine Serum (FBS)	Sigma-Aldrich	CAT#F2442
MACS BSA stock solution	Miltenyi	CAT#130-091-376
autoMACS Rinsing Solution	Miltenyi	CAT#130-091-222
Penicillin-Streptomycine-Glutamine (P/S/G)	Gibco	CAT#10378016
MEM non-essential amino acids (NEAA)	Gibco	CAT#11140050
HEPES Buffer Solution	Gibco	CAT#15630056
Sodium Pyruvate	Gibco	CAT#11360070
β-Mercaptoethanol for cell culture	Sigma-Aldrich	CAT#M3148
Normocin	Invivogen	CAT#ant-nr-2
4′,6-Diamidino-2-Phenylindole, Dilactate (DAPI)	BioLegend	CAT#422801
Fixable Viability Dye eFluor506	affymetrix eBioscience	CAT#65-0866-14
Cell Fixation/Permeabilization Kit	BD Biosciences	CAT#554714
CellTracker™ Red CMTPX	Invitrogen	CAT#C34552
Polybrene infection/transfection reagent	Millipore	CAT#TR-1003-G
Percoll	Sigma-Aldrich	CAT#P4937
Isoflurane	Zoetis	CAT#50019100
Phosphate Buffered Saline (PBS) pH 7.4 (1×)	Gibco	CAT#10010-023
Pierce Bovine Serum Albumin Standard	Thermo Fisher Scientific	CAT#23210
RIPA Lysis and Extraction Buffer	Thermo Fisher Scientific	CAT#89900
Restore Western Blot Stripping Buffer	Thermo Fisher Scientific	CAT#21059
Protease/Phosphatase Inhibitor Cocktail	Cell Signaling	CAT#5872S
4–15% Mini-PROTEAN® TGX™ Precast Protein Gels	Bio-Rad	CAT#4561084
2-mercaptoethanol	Bio-Rad	CAT#1610710
4× Laemmli Sample Buffer	Bio-Rad	CAT#1610747
Trans-Blot Turbo 5× Transfer Buffer	Bio-Rad	CAT#10026938
10× Tris/Glycine/SDS Buffer	Bio-Rad	CAT#1610772
Precision Plus Protein Dual Color Standards	Bio-Rad	CAT#1610394
Blotting Grade Blocker Non Fat Dry Milk	Bio-Rad	CAT#1706404XTU
Tween 20	Amresco	CAT#M147-1L
Dimethyl sulfoxide (DMSO)	VWR	CAT#0231-500 ML
TRIzol Reagent	Invitrogen	CAT#15596018
SsoAdvanced Universal SYBR Green Supermix	Bio-Rad	CAT#1725271
GolgiStop™ Protein Transport Inhibitor	BD Biosciences	CAT#554724
Recombinant mouse GM-CSF	Peprotech	CAT#315-03
Recombinant mouse FLT3-Ligand	Peprotech	CAT#250-31L
Recombinant human IL-2	Peprotech	CAT#200-02

**Critical commercial assays**		

Mouse CD8 T cell Isolation Kit	Miltenyi Biotec	CAT#130-104-075
Trans-Blot Turbo RTA Mini 0.2 μm PVDF Transfer Kit	Bio-Rad	CAT#1704272
Cytiva Amersham ECL Prime Western Blotting Detection Kit	Cytiva	CAT#RPN2232
Fixation/Permeabilization Solution Kit	BD Sciences	CAT#554714
Bicinchoninic Acid (BCA) Assay Kit	Thermo Fisher Scientific	CAT#23228 and 1859078
Nuclear Protein Extraction Kit	Thermo Fisher Scientific	CAT#P178833
SuperScript III First-Strand Synthesis Supermix Kit	Invitrogen	CAT#18080400
Chromium Single Cell 3′ Library & Gel Bead Kit v2	10× Genomics	CAT#PN-120237
NovaSeq 6000 S2 Reagent Kit	Illumina	CAT#20012862

**Deposited data**		

scRNA-seq of mouse melanoma TIIs (control and creatine-treated)	Gene Expression Omnibus	GSE303921
LC-MS/MS of control and creatine-treated *CrT*-WT/KO BMDCs	MetaboLights	MTBLS12797

**Experimental models: Cell lines**		

Mouse melanoma cell line B16-OVA	Provided by Dr. Pin Wang (University of Southern California)	–
Human embryonic kidney 293T cell line	ATCC	CAT# CRL-3216; RRID: CVCL-0063

**Experimental models: Organisms/strains**		

C57BL/6J (B6) mouse	The Jackson Laboratory	Strain #:000664 RRID:IMSR_JAX:000664
B6.SJL-*Ptprc*^*a*^*Pepc*^*b*^/BoyJ (CD45.1) mouse	The Jackson Laboratory	Strain #:002014 RRID:IMSR_JAX:002014
B6(Cg)-*Slc6a8*^tm1.2Clar^/J (*CrT*-KO) mouse	The Jackson Laboratory	Strain #:021072 RRID:IMSR_JAX:021072
*OT1*-Tg mouse	This paper	–

**Recombinant DNA**		

Vector: parental pMSGV vector	This paper	–

**Oligonucleotides**		

Primers for RT-qPCR	Table S1	Table S1

**Software and algorithms**		

FlowJo	BD Biosciences	https://www.flowjo.com/solutions/flowjo
Biorender	Biorender	https://www.biorender.com
Photoshop	Adobe	https://www.adobe.com/products/photoshop
I-control 1.7 Microplate Reader Software	Tecan	https://www.selectscience.net/tecan/i-control–microplate-reader-software/81307
ImageJ	NIH	https://imagej.net
Graphpad Prism 9	Graphpad	https://www.graphpad.com/scientific-software/prism
R	R Consortium	http://www.R-project.org
RStudio	RStudio	https://posit.co
MetaboAnalyst 6.0	University of Alberta	https://www.metaboanalyst.ca/
ShinyGO 0.77	South Dakota State University	https://bioinformatics.sdstate.edu/go77

### Experimental model and study participant details

#### Mice

C57BL/6J (B6), B6.SJL-*Ptprc*^*a*^*Pepc*^*b*^/BoyJ (CD45.1), C57BL/6-Tg (TcraTcrb)1100Mjb/J (*OT1*-Tg), and B6(Cg)-*Slc6a8*^tm1.2Clar^/J (*CrT*-KO) were purchased from the Jackson Laboratory (JAX; Bar Harbor). *CrT*-KO mice were backcrossed with C57BL/6J mice for more than six generations at the University of California, Los Angeles (UCLA). All animals were maintained in the animal facilities at UCLA. Eight-to twelve-week-old male mice were used for all experiments unless otherwise indicated. All animal experiments were approved by the Institutional Animal Care and Use Committee of UCLA (Protocol #ARC-2013-054-AM-011).

#### Tumor cell lines

The B16-OVA mouse melanoma cell line was kindly provided by Dr. Pin Wang (University of Southern California, CA, USA). This tumor cell line was cultured in D10 medium at 37°C and with 5% CO_2_. All tumor cell lines utilized in this study underwent short tandem repeat (STR) profiling, and the resulting profiles were compared to established databases to confirm accurate identification. Furthermore, the cell lines were regularly screened for mycoplasma contamination to preserve their integrity and authenticity.

#### Media and reagents

DMEM-based adherent cell culture medium (denoted as D10 medium) was made of Dulbecco’s modified Eagle’s medium (DMEM; catalog no. 10013, Corning) supplemented with 10% fetal bovine serum (FBS; catalog no. F2442, Sigma-Aldrich) and 1% penicillin-streptomycin-glutamine (catalog no. 10378016, Gibco). RPMI-based immune cell culture medium (denoted as C10 medium) was made of RPMI 1640 (catalog no. 10040, Corning) supplemented with 10% FBS (catalog no. F2442, Sigma-Aldrich), 1% penicillin-streptomycin-glutamine (catalog no. 10378016, Gibco), 0.2% Normocin (catalog no. ant-nr-2, InvivoGen), 1% Minimal Essential Medium (MEM) Non-essential Amino Acid Solution (catalog no. 11140050, Gibco), 1% HEPES (catalog no. 15630080, Gibco), 1% sodium pyruvate (catalog no. 11360070, Gibco), and 0.05 mM beta-mercaptoethanol (catalog no. M3148, Sigma-Aldrich). Bone marrow-derived dendritic cell (BMDC) culture medium was made of C10 medium with 20 ng/mL murine GM-CSF (catalog no. 315-03, Thermo Fisher Scientific). Bone marrow-derived conventional dendritic cell 1 (BMDC1) culture medium was made of C10 medium with 2 ng/mL murine GM-CSF and 50 ng/mL murine FLT3L (catalog no, 250-31L, Peprotech). Monocyte-derived dendritic cell (MoDC) culture medium was made of C10 medium with 20 ng/mL human GM-CSF (catalog no. G5035, Sigma-Aldrich) and 20 ng/mL human IL-4 (catalog no. 200-04, Thermo Fisher Scientific). Dialyzed C10 medium was made of C10 with 10% dialyzed FBS (catalog no. A3382001, Fisher) instead of FBS.

Anti-human CD3 (catalog no. 300314, clone HIT3a) and anti-human CD28 (1 mg/mL; catalog no. 302902, clone CD28.2) were purchased from BioLegend. Recombinant human IL-2 (catalog no. 200-02) was purchased from PeproTech.

Creatine monohydrate (catalog no. C3630) was purchased from Sigma-Aldrich. A creatine transporter inhibitor, RGX-202 (catalog no. HY-W015828), was purchased from MedChemExpress.

### Method details

#### Syngeneic mouse tumor models

B16-OVA melanoma cells (1 × 10^6^ per animal) were subcutaneously injected into the right flanks of experimental mice to form solid tumors. For creatine treatment experiments, mice received intraperitoneal injection of creatine (10.5 mg per animal per day) dissolved in sterile 0.9% saline. Throughout the course of experiments, tumor size was measured twice per week by using a Fisherbrand Traceable digital caliper (Thermo Fisher Scientific). Tumor volumes were calculated by formula 1/2 × L × W^2^. At the end of an experiment, tumor-infiltrating immune cells (TIIs) were isolated for analysis using qPCR, flow cytometry, and/or scRNA-seq.

#### Tumor-infiltrating immune cell (TII) *ex vivo* analysis

Solid tumors were harvested from experimental mice and mechanically meshed in 70-mm cell strainers (catalog no. 07-201-431, Corning) to generate single cell suspensions. Single cells were washed once with C10 medium, resuspended in 50% Percoll (catalog no. P4937, Sigma-Aldrich), and centrifuged at 800 × g at 25°C for 30 min with brake off. Cell pellets enriched with TIIs were then collected for further analysis.

In the experiments studying gene expression in tumor-infiltrating DCs, day 14 B16-OVA tumors were harvested from B6 wild-type mice to prepare TII suspensions. Tumor-infiltrating DCs (gated as DAPI^−^CD45.2^+^ CD11c^+^I-A^bhi^ cells) were sorted using a FACSAria II flow cytometer and then were subjected to qPCR analysis.

In the experiments studying gene expression profiling of TIIs, day 10 B16-OVA tumors were harvested from creatine-treated mice to prepare TII suspensions. TII suspensions were then sorted using a FACSAria II flow cytometer to purify immune cells (gated as DAPI^−^CD45.2^+^ cells) that were subjected to scRNA-seq analysis.

In the experiments studying status and function of tumor-infiltrating DCs, TII suspensions were prepared and then analyzed by flow cytometry to study their expression of surface activation markers.

#### *In vitro* mouse BMDC culture

To generate BMDCs, BM cells were collected from femurs and tibias of *CrT*-WT mice and *CrT*-KO mice and were cultured in BMDC culture medium in a 10-cm dish (0.2 × 10^6^ cells per mL; 10 mL per dish) for 9 days. At day 3, 10 mL BMDC culture medium was added, and at day 5, 10 mL medium was replaced. At day 9, the resulting BMDCs were collected and reseeded in a 24-well plate (0.5 × 10^6^ cells per mL; 1 mL per well) in C10 medium for 24h, in the presence or absence of LPS (10–100 ng/mL) (catalog no. L5293, Sigma-Aldrich) or poly(I:C) (10 μg/mL) (catalog no. P1530, Sigma-Aldrich) to stimulate BMDCs.

In some experiments, creatine (0.5 mM) or ATP (0.5 μM) was added to the *CrT*-WT BMDC culture 30 min prior to adding LPS stimulation. In some experiments, creatine transporter inhibitor (RGX-202) (20 μM) was added 24h prior to LPS stimulation. Unless otherwise noted, BMDCs were collected at 24h for RT-qPCR analysis and at 72h for flow cytometry analysis.

#### *In vitro* mouse BMDC1 culture

To generate BMDC1s, BM cells were collected from femurs and tibias of *CrT*-WT mice and were cultured in BMDC1 culture medium in a 10-cm dish (1.5 × 10^6^ cells per mL; 10 mL per dish) for 13 days. At day 5, 10 mL BMDC culture medium was added, and at day 9, lightly adherent cells were collected via centrifugation and replated in 10 mL medium. At day 13, the resulting BMDC1s were collected and reseeded in a 96-well plate (0.25 × 10^6^ cells per mL; 0.2 mL per well) for subsequent analysis.

#### BMDC adoptive transfer

*CrT*-WT or *CrT*-KO BMDCs were stimulated with LPS (100 ng/mL) for 24h and loaded with ovalbumin peptide 1 (OVAp1) (0.1 μg/mL) for an additional hour. 2 × 10^6^ BMDCs from each group are s.c. injected into BoyJ mice. At day 7, spleens were harvested from mice and seeded in a 96-well plate (5 × 10^6^ splenocytes per mL, 0.2 mL per well) and stimulated with OVAp1 (0.1 μg/mL). Supernatant was collected at 24h for ELISA analysis of inflammatory cytokines.

#### Mouse OT1 T:BMDC coculture

Spleen and lymph node cells were harvested from the *OT1*-Tg mice and were subjected to magnetic-activated cell sorting (MACS) using a Mouse CD8 T cell Isolation Kit (catalog no. 130-104-075, Miltenyi Biotec) following the manufacturer’s instructions. For some experiments, the OT1 T cells were stained with CellTracker CMPTX cell division dye (catalog no. C34552, Invitrogen) according to the manufacturer’s instructions for 15 min. The purified OT1 T cells (identified as CD8^+^ TCR Vβ5^+^ cells) were cocultured with BMDCs and OVAp1 (0.1 μg/mL) (catalog no. RP10611, GenScript) in a 24-well plate at a DC:T cell ratio of 1:10 (0.05 × 10^6^ BMDCs per mL, 1 mL per well). Samples were taken every 24h for T cell counting via flow cytometry. Activation of OT1 T cells was analyzed at 96 h by measuring T cell expression of surface markers CD25, CD69, and CD44, and intracellular cytokines IL-2 and IFN-γ using flow cytometry.

#### *In vitro* human MoDC culture

To generate MoDCs, CD14^+^ cells were isolated from HLA-A2^+^ donor PBMCs using MACS sorting with human CD14 MicroBeads (catalog no. 130-050-201, Miltenyi Biotec) according to the manufacturer’s instructions. The purified CD14^+^ cells were cultured in MoDC culture medium (1 × 10^6^ cells per mL; 10 mL per dish) for 5 days. At day 3, 10 mL of MoDC culture medium was added to the culture. At day 5, the resulting MoDCs were collected and reseeded in a 96-well plate (2.5 × 10^5^ cells per mL, 0.2 mL per well) in C10 medium for 24h, in the presence or absence of recombinant human TNF-α (50 ng/mL) (catalog no. 300-01A, Thermo Fisher Scientific) to stimulate MoDCs.

In some experiments, creatine was added to the MoDC culture 24h prior to adding TNF-α stimulation. At 24h after LPS stimulation, MoDCs were collected for analysis.

#### Human ESO-T:MoDC coculture

Healthy donor PBMC-derived NY-ESO-1 specific human T lymphocytes (ESO-T cells) were generated through stimulating human T cells with anti-human CD3 and CD28 antibodies for 2 days, engineering these cells with a lentivector encoding a 1G4 TCR^38^ (HLA-A2-restricted, NY-ESO-1 tumor antigen-specific), and culturing in C10 medium supplemented with human IL-2 (10 ng/mL) for 2 weeks. TNF-α-stimulated MoDCs were reseeded in a 6-well plate (5 × 10^6^ cells per mL, 2 mL per well) at loaded with NY-ESO-1 peptide (0.5 μM) (catalog no. 6-7013-901, IBA Lifesciences) with agitation every 30 min. ESO-T cells and NY-ESO-1 peptide-loaded MoDCs were cocultured in a 24-well plate at a ratio of 1:1 (0.1 × 10^6^ MoDCs per mL, 1 mL per well). Activation of ESO-T cells was analyzed at 24 h by measuring T cell surface expression of CD25, CD69, and CD62L using flow cytometry.

#### Flow cytometry

Flow cytometry was used to analyze the expression of surface markers of DCs and T cells. Fluorochrome-conjugated monoclonal antibodies specific for mouse CD45.2 (clone 104), CD11b (clone M1/70), CD11c (clone RM4-5), CD103 (clone 2E7), S8:H2-K^b^ (clone 25-D1.16), I-A^b^ (clone AF6-120.1), CD80 (clone 16-10A1), CD86 (clone GL-1), TCRβ (clone H57-597), CD8 (clone 53–6.7), CD69 (clone H1.2F3), CD25 (clone PC61), CD44 (clone IM7), CD62L (clone MEL-14), IL-2 (clone JES6-5H4), IFN-γ (clone XMG1.2), XCR1 (clone ZET), CD14 (clone Sa14-2), TNF-α (clone MP6-XT22), IL-12p40 (clone C15.6), IL-6 (clone MP5-20F3) were purchased from BioLegend. Fc block (anti-mouse CD16/32) (clone 2.4G2) was purchased from BD Biosciences. Fluorochrome-conjugated monoclonal antibodies specific for human CD45 (clone HI30), CD3 (clone OKT3), CD4 (clone OKT4), CD8 (clone SK1), TCR Vβ13.1 (clone H131), CD62L (clone DREG-56), CD69 (clone FN50), CD25 (clone M-A251) were purchased from BioLegend. Human Fc Receptor Blocking Solution (catalog no. 422302) was purchased from BioLegend. Fixable Viability Dye eFluor 506 (catalog no. 65–0866) was purchased from Thermo Fisher Scientific.

Cells were initially stained with a Fixable Viability Dye, followed by Fc blocking and surface marker staining, as described previously. To detect intracellular molecules, cells were pretreated with a protein transport inhibitor (catalog no. 554724, BD Biosciences) 4 to 24 h before collection and subjected to intracellular staining using a Cell Fixation/Permeabilization Kit (catalog no. 554714, BD Biosciences) following the manufacturer’s instructions. Stained cells were analyzed using a MACSQuant Analyzer 10 Flow Cytometer (Miltenyi Biotec). FlowJo 10 software (Tree Star) was used to analyze the data.

#### mRNA quantitative RT-PCR

Total RNA was isolated using TRIzol Reagent (catalog no. 15596018, Thermo Fisher Scientific) and the miRNeasy Mini Kit (catalog no. 217004, QIAGEN) according to the manufacturers’ instructions. cDNA was prepared using the SuperScript III First-Strand Synthesis Supermix Kit (catalog no. 18080400, Thermo Fisher Scientific). Gene expression was measured using the SsoAdvanced Universal SYBR Green Supermix (catalog no. 1725271, Bio-Rad) and the 7500 Real-time PCR System (Applied Biosystems) according to the manufacturers’ instructions. *Ube2d2* was used as an internal control for mouse DCs. *BACTIN* was used as an internal control for human MoDCs. The relative expression of the mRNA of interest was calculated using the 2^ΔΔCT^ method and is presented as the fold induction relative to the control. Primer sequences are shown in Table S1.

#### ELISA

ELISA for detecting mouse cytokines were performed following a standard protocol from BD Biosciences. Capture and biotinylated antibody pairs for the detection of mouse IFN-γ (coating antibody, catalog no. 554424; biotinylated detection antibody, catalog no. 554426), IL-2 (coating antibody, catalog no. 551216; biotinylated detection antibody, catalog no. 554410), and TNF-α (coating antibody, catalog no. 14-7325-81; biotinylated detection antibody, catalog no. 506312) were purchased from BD Biosciences (IFN-γ and IL-2) or Invitrogen and Biolegend (TNF-α). The streptavidin–horseradish peroxidase (HRP) conjugate (catalog no. 18410051) was purchased from Invitrogen. Mouse IFN-γ (catalog no. 575309) and IL-2 (catalog no. 575409) standards were purchased from BioLegend. Samples were analyzed for absorbance at 450 nm using an Infinite M1000 microplate reader (Tecan).

#### Western blots

BMDCs from *Crt*-WT or *CrT*-KO mice were cultured *in vitro* in a 24-well plate at 0.5 × 10^6^ cells per well for 2 days, in the presence of LPS (100 ng/mL). In some experiments, creatine (0.5 mM) or ATP (0.5 μM) was added to the *CrT*-WT BMDC culture 30 min prior to adding LPS stimulation. In signal transduction experiments, creatine (1 mM) and creatine transporter inhibitor (RGX-202) (20 μM) was added 24h prior to LPS stimulation. Total protein was extracted using a RIPA lysis and extraction buffer (catalog no. 89900, Thermo Fisher Scientific) supplemented with protease/phosphatase inhibitor cocktail (catalog no. 5872S, Cell Signaling Technology). Nuclear protein was extracted using the Nuclear Protein Extraction Kit (catalog no. P178833, Thermo Fisher Scientific). Protein concentration was measured using the Bicinchoninic Acid (BCA) Assay Kit (catalog nos. 23228 and 1859078, Thermo Fisher Scientific). Equal amounts of protein were resolved on a 10% SDS-polyacrylamide gel electrophoresis gel and then transferred to a polyvinylidene difluoride (PVDF) membrane by electrophoresis. The following antibodies were purchased from the Cell Signaling Technology and used to blot for the proteins of interest: anti-mouse NF-κB p65 (catalog no. 8242S, clone D14E12), anti-mouse c-Jun (catalog no. 9165S, clone 60A8), anti-mouse NFAT (catalog no. 4389S), anti-mouse phospho-IκBα (catalog no. 2859, clone 14D4), anti-mouse IκBα (catalog no. 4814, clone L35A5), anti-mouse phospho-IKKα/β (catalog no. 2697, clone 16A6), anti-mouse IKKβ (catalog no. 8943, clone D30C6), anti-mouse phospho-NF-κB p65 (catalog no. 3033, clone 93H1), secondary anti-mouse (catalog no. 7076P2), and secondary anti-rabbit (catalog no. 7074P2). GAPDH (catalog no. 2118S, clone 14C10, Cell Signaling Technology) was used as an internal control for cytoplasmic proteins, whereas Lamin A/C (catalog no. 39287, clone 3A6-4C11, Active Motif) was used as an internal control for nuclear proteins. Signals were visualized with autoradiography using an enhanced chemiluminesence (ECL) prime western blotting system (catalog no. RPN2232, Cytiva). Data analysis was performed using ImageJ software (NIH).

#### Liquid chromatography-tandem mass spectrometry (LC-MS/MS)

*CrT*-WT or *CrT*-KO BMDCs were stimulated with 100 ng/mL LPS for 24 h in complete C10 medium. *CrT*-WT BMDCs were stimulated with 100 ng/mL LPS for 24 h with or without creatine supplementation in dialyzed C10 medium. Metabolites were extracted using the methanol/chloroform/water method as previously described.^60^ Dried metabolites were resuspended in 100 μL 50% acetonitrile:water and 5 μL was loaded onto a Luna NH_2_ 3 μm 100 Å (150 × 2.0 mm) column (Phenomenex) using a Vanquish Flex UHPLC system (Thermo Scientific). The chromatographic separation was performed with mobile phases A (5 mM NH_4_AcO, pH 9.9) and B (acetonitrile) at a flow rate of 200 μL/min. A linear gradient from 15% A to 95% A over 18 min was followed by 7 min isocratic flow at 95% A and reequilibration to 15% A. Metabolites were detected with a Thermo Scientific Q Exactive mass spectrometer run with polarity switching in full scan mode using a range of 70–975 m/z and 70.000 resolution. Maven (v 8.1.27.11) was used to quantify the targeted polar metabolites by AreaTop, using expected retention time and accurate mass measurements (<5 ppm) for identification.

#### Single-cell RNA sequencing (scRNA-seq)

scRNA-seq was used to analyze the gene expression profiling of TIIs. Day 14 B16-OVA tumors were harvested from experimental mice to prepare TII suspensions (4 tumors were combined for each group). TII suspensions were then sorted using a FACSAria II flow cytometer to purify immune cells (gated as DAPI^−^CD45.2^+^ cells). Sorted TIIs were immediately delivered to the Technology Center for Genomics & Bioinformatics (TCGB) facility at UCLA for library construction and sequencing. Briefly, purified TIIs were quantified using a Cell Countess II automated cell counter (Invitrogen/Thermo Fisher Scientific). A total of 10,000 TIIs from each experimental group were loaded on the Chromium platform (10× Genomics), and libraries were constructed using the Chromium Single Cell 3′Library & Gel Bead Kit v2 (catalog no. PN-120237, 10× Genomics) according to the manufacturer’s instructions. Libraries were sequenced on an Illumina NovaSeq using the NovaSeq 6000 S2 Reagent Kit (100 cycles; catalog no. 20012862, Illumina).

### Quantification and statistical analysis

#### Metabolomics data analysis

MRM Q1/Q3 peak integration of the raw data files was analyzed with software Sciex OS 2.0 (ABSciex). A total of 157 metabolites were identified. The abundance of each metabolite was indicated by the peak area and normalized to the bookended pooled tissue extracts. Data analysis, including principal component analysis and heatmap generation was performed using in-house R scripts. Pathway enrichment analyses were conducted using Metaboanalyst 6.0 (https://www.metaboanalyst.ca/) using the Small Molecule Pathway Database (SMPDB) reference database.

#### Sequencing data analysis

Data analysis was performed using a Cell Ranger Software Suite (10× Genomics). Binary base call (BCL) files were extracted from the sequencer and used as inputs for the Cell Ranger pipeline to generate the digital expression matrix for each sample. Then, the Cell Ranger aggr command was used to aggregate the two samples into one digital expression matrix. The matrix was analyzed using Seurat (v.5.3.0), an R package designed for scRNA-seq. Specifically, cells were first filtered to have at least 300 unique molecular identifiers (UMIs), at least 100 genes, and at most 25% mitochondrial gene expression; only one cell did not pass the filter. The filtered matrix was normalized using the Seurat function NormalizeData through natural-log transformation. Variable genes were found using the Seurat function FindVariableGenes and ScaleData function was used to regress out the sequencing depth for each cell. Variable genes that had been previously identified were used in principle components analysis (PCA) to reduce the dimensions of the data. The Seurat integration pipeline was performed following the Seurat guidelines. After this, 30 principal components (PCs) were used in Uniform Manifold Approximation and Projection (UMAP) to further reduce the dimensions to two. The same 30 PCs were also used to group the cells into different clusters by the Seurat function FindClusters. Next, marker genes were found for each cluster using the FindAllMarkers function and used to define the cell types. Cell types were manually annotated based on the cluster markers. To perform comparative scRNA-seq analysis of across experimental conditions between PBS and creatine, the clusters consisting of tumor-infiltrating cDCs (identified by co-expression of marker genes) were extracted using Subset function. For pathway analysis, the fold enrichment value was calculated as following: Fold_enrichment = (N intersect/N DEG)/(N pathway/N background), where N intersect indicates the number of genes from differentially expressed gene set that are present in the pathway gene set; N DEG indicates number of genes from differentially expressed gene set; N pathway indicates the number of genes from the pathway gene set; and N background indicates the total number of genes in this analysis.

#### Statistics

GraphPad Prism 9 software (GraphPad Software) was used for statistical data analysis. Pairwise comparisons were made using a two-tailed Student’s t test. Multiple comparisons were performed using an ordinary one-way analysis of variance (ANOVA) followed by Tukey’s multiple comparisons test or a two-way repeated measures ANOVA followed by Sidak multiple comparisons test. Data are presented as the means ± SEM, unless otherwise indicated. A *p* value of less than 0.05 was considered significant. ns, not significant; ∗*p* < 0.05, ∗∗*p* < 0.01, ∗∗∗*p* < 0.001 and ∗∗∗∗*p* < 0.0001. The *p* values of violin plots were determined by the Wilcoxon test.
