# Supplementary material for: Creatine uptake promotes dendritic cell activation and enhances antitumor immunity
Source: iScience. 2026 Mar 21;29(4):115436. doi: 10.1016/j.isci.2026.115436 (PMC13091023; doi:10.1016/j.isci.2026.115436)
Supplement: Document S1. Figures S1–S8 and Table S1 [file mmc1.pdf]

iScience, Volume *29*

Supplemental information

Creatine uptake promotes dendritic cell activation and enhances antitumor immunity

Elliot Kang, James Elsten-Brown, Yu-Chen Wang, Ashley Lam, Elise Sanchez, Renee Wen, Tiffany Wang, Jennifer Chiang, Quentin Scarborough, Yan-Ruide Li, Yichen Zhu, Jie Huang, Matthew Williams, Sarah Eckl, Bo Li, and Lili Yang

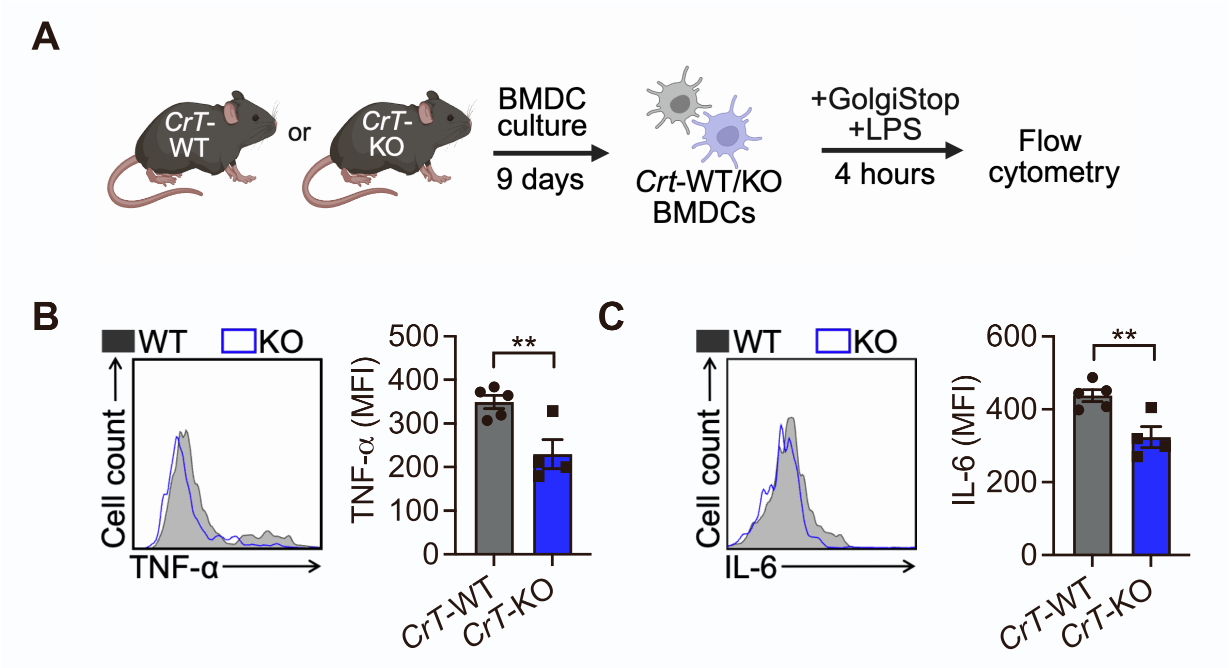

Figure S1. Creatine uptake deficiency and BMDC cytokine production, related to Figure 1

(A) Experimental design. (B) FACS analysis of intracellular TNF-α (B) and IL-6 (C) production in *CrT*-WT and *CrT*-KO BMDCs treated with 100 ng/ml LPS at 4 hours post-stimulation (n = 4-5).

Data are presented as the mean ± SEM. $^{**}p < 0.01$ by Student's t-test.

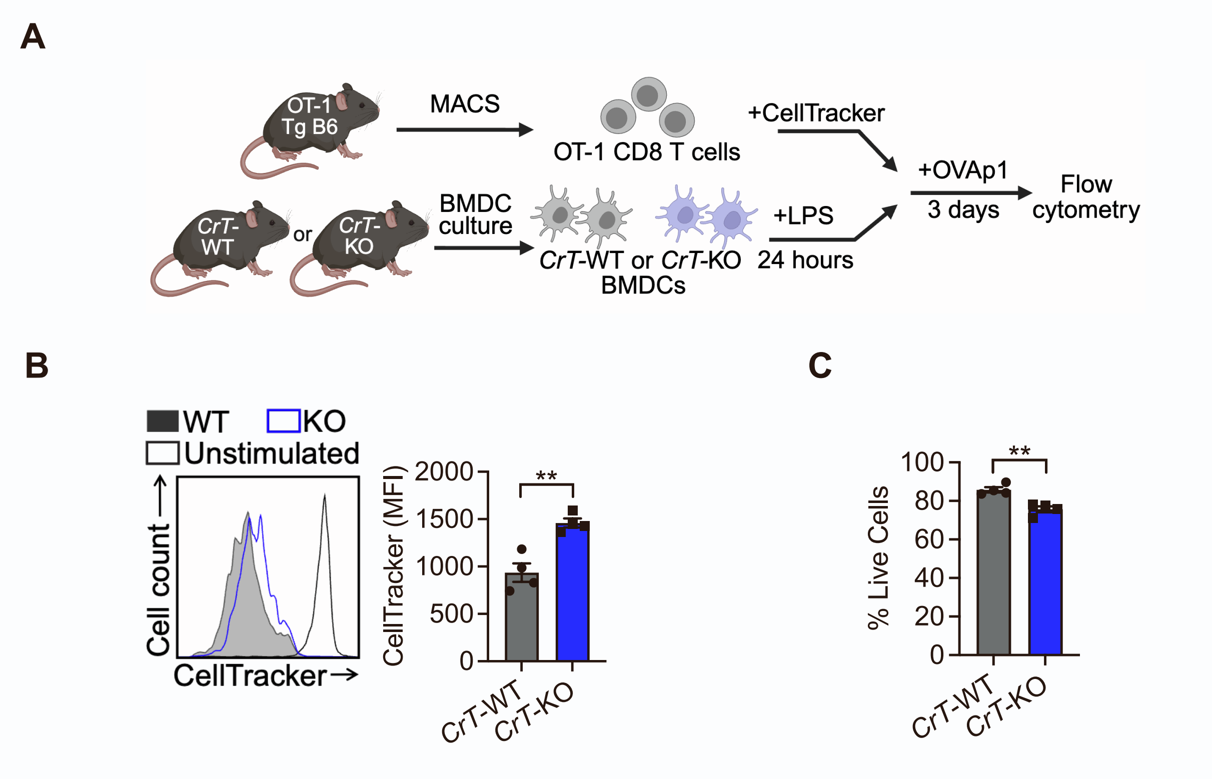

Figure S2. Creatine uptake deficiency and dendritic cell-mediated T cell division, related to Figure 2

(A) Experimental design. *CrT*-WT or *CrT*-KO LPS-activated (100 ng/ml) BMDCs loaded with OVAp1 (0.1 μg/ml) were cocultured with CD8 T cells isolated from OT1-Tg mice. T cells were stained with a cell division tracker. (B-C) FACS analysis of cell division tracker (B) and viability (C) in CD8 T cells at day 3 ($n = 4$).

Data are presented as the mean ± SEM. $^{**}p < 0.01$ by Student's t-test.

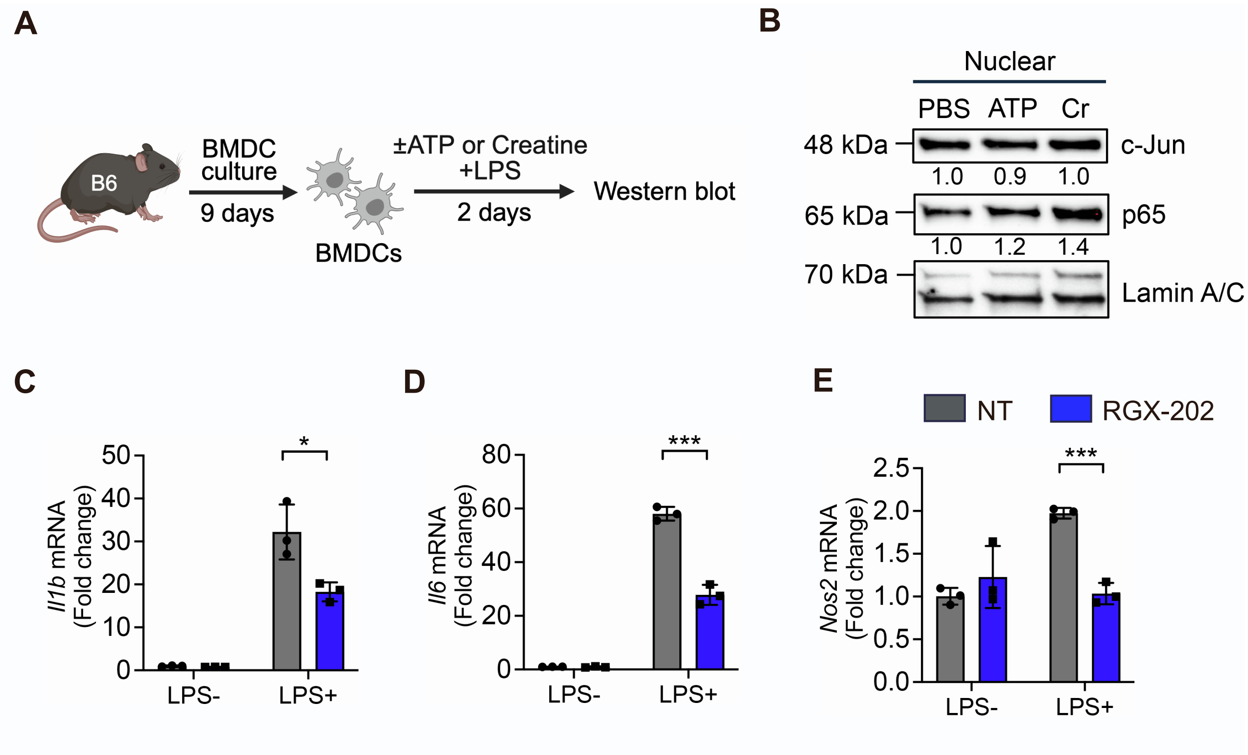

Figure S3. RGX-202 inhibitor studies, related to Figure 4

(A-B) Creatine or ATP treatment experiment. (A) Experimental design. (B) Western blot analysis of DC activation transcription factors in control, ATP- and creatine-treated BMDCs.

(C-E) Phenotype analysis of RGX-202-treated BMDCs. RT-qPCR analysis of inflammatory markers *Il1b* (C), *Il6* (D), and *Nos2* (E) in BMDCs treated with or without 20 μM creatine transporter inhibitor RGX-202 24 hours before LPS stimulation (n = 3).

Representative of two (B) and three (C-E) experiments. Data are presented as the mean ± SEM. $^{*}p < 0.05$ and $^{***}p < 0.001$ by Student's t-test.

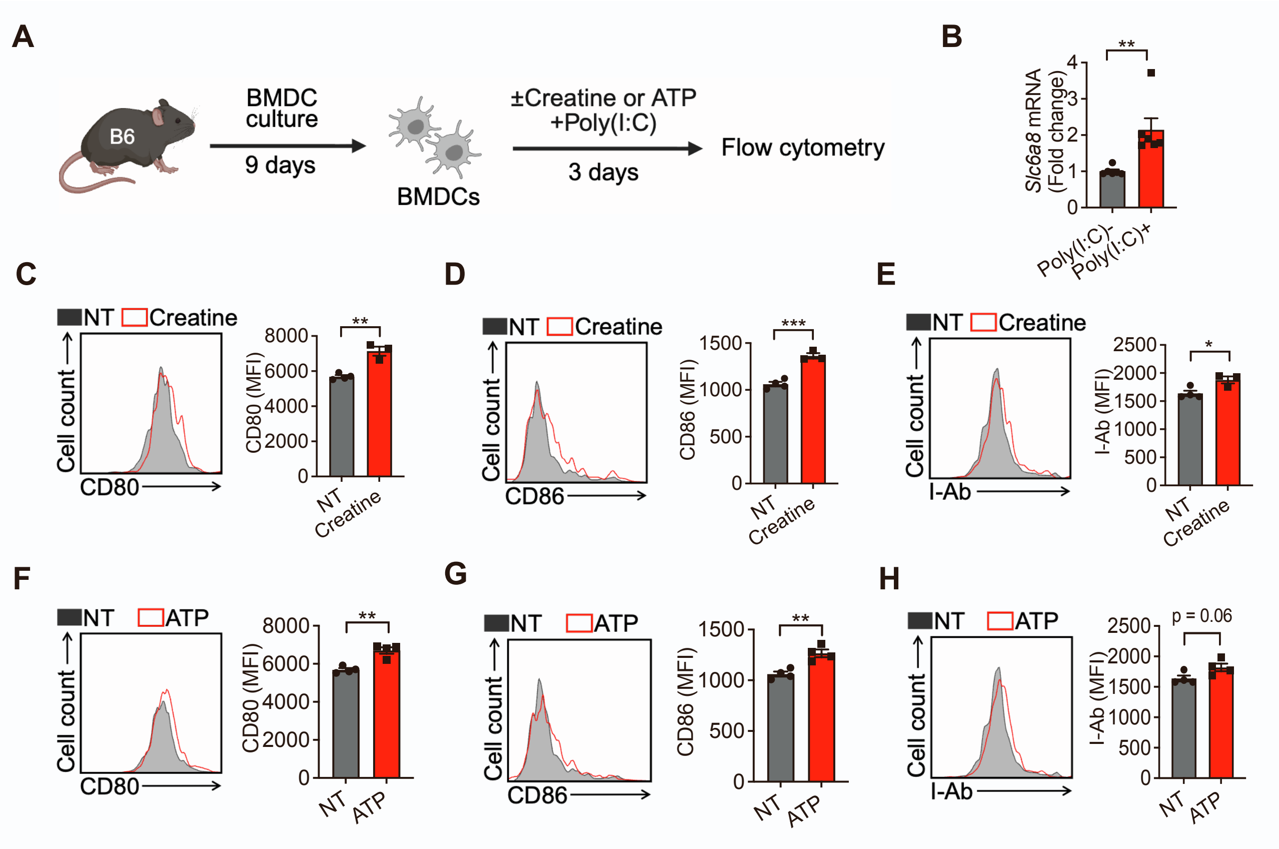

Figure S4. Creatine uptake and BMDC TLR3 responses, related to Figure 4

(A) Experimental design. (B) RT-qPCR analysis of *CrT* mRNA expression in BMDCs treated with 10 μg/ml poly(I:C) (n = 6). Unstimulated cells were included as a control (n = 6).

(C-E) BMDC activation under creatine treatment. FACS analyses of CD80 (C), CD86 (D), and I-Ab (E) levels in BMDCs treated with 0.5 mM creatine and 10 μg/ml poly(I:C) at day 3 (n = 3-4).

(F-H) FACS analyses of CD80 (F), CD86 (G), and I-Ab (H) levels in BMDCs treated with 0.5 μM ATP and 10 μg/ml poly(I:C) at day 3 (n = 4).

Data are presented as the mean ± SEM. *$p < 0.05$, **$p < 0.01$, and ***$p < 0.001$ by Student's t-test.

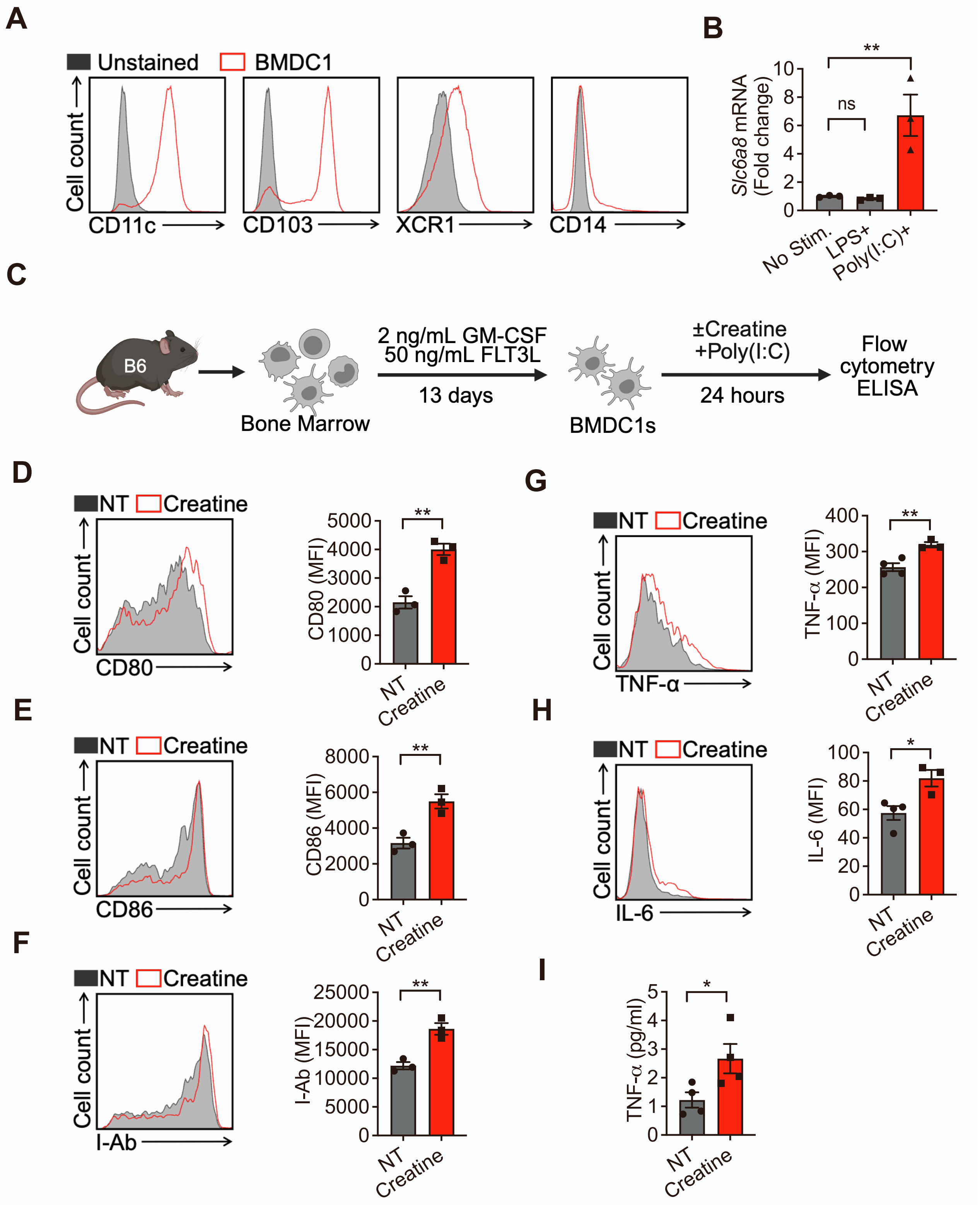
A
Unstained
BMDC1
Cell count
CD11c
CD103
XCR1
CD14
B
Slc6a8 mRNA (Fold change)
10
8
6
4
2
0
**
ns
No Stim.
LPS+
Poly(I:C)+
C
B6
Bone Marrow
2 ng/mL GM-CSF
50 ng/mL FLT3L
13 days
BMDC1s
±Creatine
+Poly(I:C)
24 hours
Flow cytometry
ELISA
D
NT
Creatine
Cell count
CD80
CD80 (MFI)
5000
4000
3000
2000
1000
0
**
NT
Creatine
E
NT
Creatine
Cell count
CD86
CD86 (MFI)
8000
6000
4000
2000
0
**
NT
Creatine
F
NT
Creatine
Cell count
I-Ab
I-Ab (MFI)
25000
20000
15000
10000
5000
0
**
NT
Creatine
G
NT
Creatine
Cell count
TNF-α
TNF-α (MFI)
400
300
200
100
0
**
NT
Creatine
H
NT
Creatine
Cell count
IL-6
IL-6 (MFI)
100
80
60
40
20
0
*
NT
Creatine
I
TNF-α (pg/ml)
5
4
3
2
1
0
*
NT
Creatine

Figure S5. Creatine uptake and BMDC1 activation, related to Figure 5

(A) FACS analysis of bone marrow-derived dendritic cell 1 (BMDC1) phenotype at day 12 of culture. (B) RT-qPCR analysis of *CrT* mRNA expression in BMDC1s treated with 100 ng/ml LPS or 10 μg/ml poly(I:C) ($n = 3$). Unstimulated cells were included as a control ($n = 3$).

(C-I) BMDC1 activation under creatine treatment. (C) Experimental design. (D-F) FACS analyses of CD80 (D), CD86 (E), and I-Ab (F) levels in BMDC1s treated with 1 mM creatine and 10 μg/ml poly(I:C) at day 3 ($n = 3$). (G-H) FACS analyses of intracellular TNF-α (G) and IL-6 (H) production in BMDC1s at 4 hours post-stimulation ($n = 3$-4). (I) ELISA analysis of TNF-α levels in BMDC1 supernatant collected 24 hours post-stimulation ($n = 4$).

Data are presented as the mean ± SEM. $^{*}p < 0.05$, $^{**}p < 0.01$ by one-way ANOVA (B) or Student's t-test (D-I).

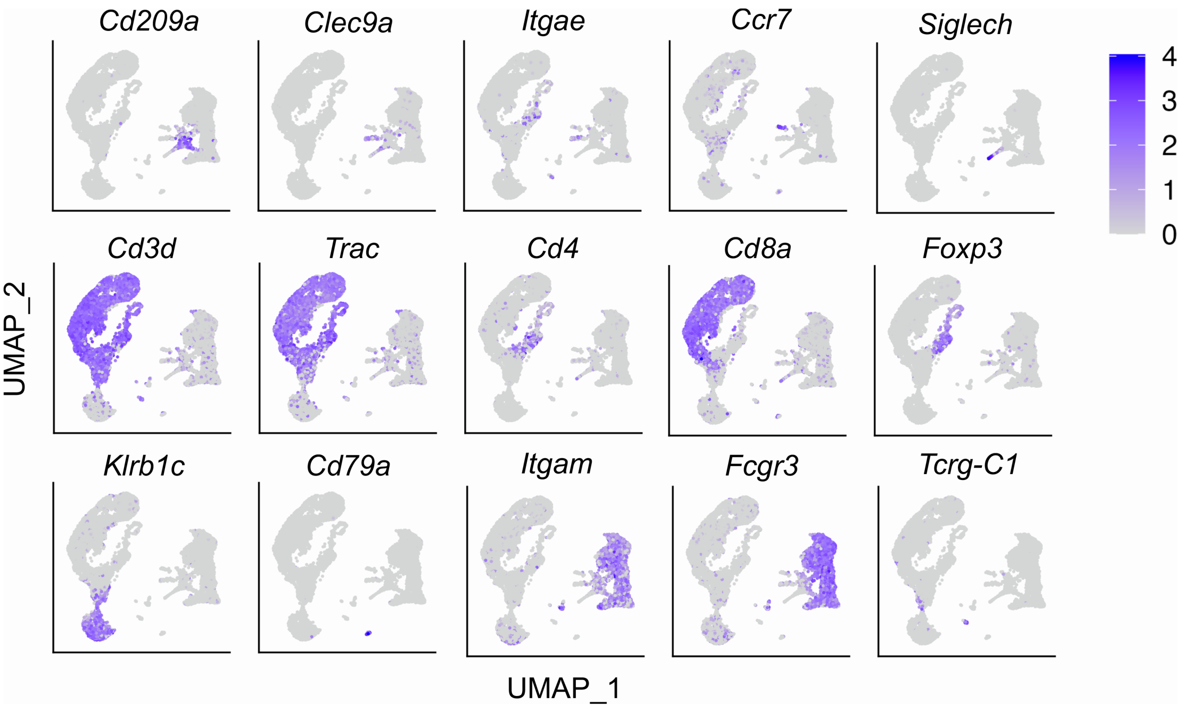

Figure S6. Cluster identification for single-cell RNA-sequencing analysis, related to Figure 5

Combined UMAP plots showing the expression patterns of 15 marker genes used to define the 10 cell clusters. Each dot represents one single cell and is colored according to its expression of the indicated marker gene.

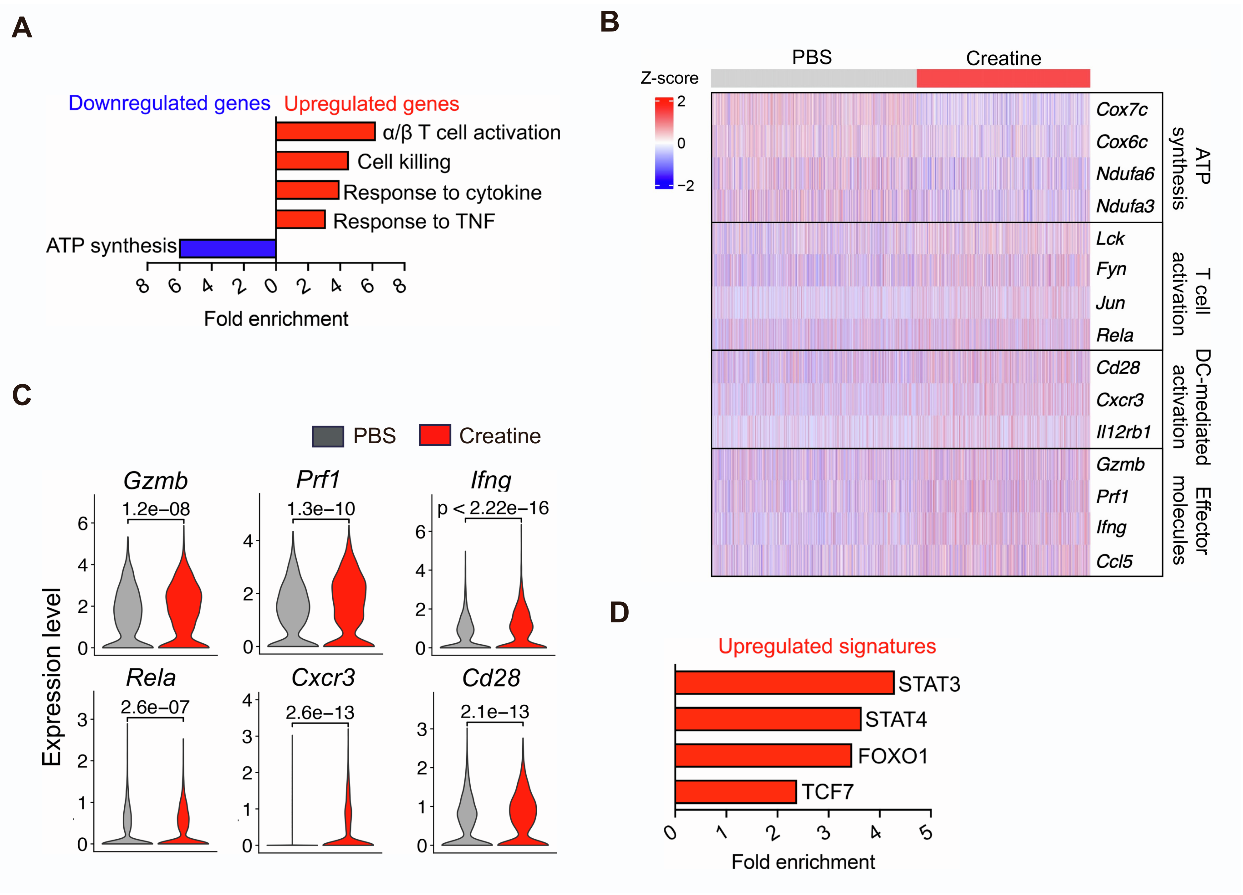

Figure S7. Creatine uptake and intratumoral CD8 T cell phenotypes, related to Figure 5

(A) Bar plot showing the fold enrichment of indicated pathways in genes upregulated or downregulated in creatine-treated CD8 T cells compared to control CD8 T cells. (B) Heatmap showing the expression of representative genes in each treatment group. Each column represents an individual cell. (C) Violin plots of key genes for CD8 T cell activation, effector function, and communication with DCs. *p* values from the Wilcoxon test are shown. (D) Transcription factor target enrichment analysis. Bar plot showing the fold enrichment of indicated transcription factor target genes in creatine-treated CD8 T cells.

Single-cell RNA sequencing was performed once, and cells isolated from 5 mice of each experimental group were combined for analysis. $p < 0.05$ was considered significant.

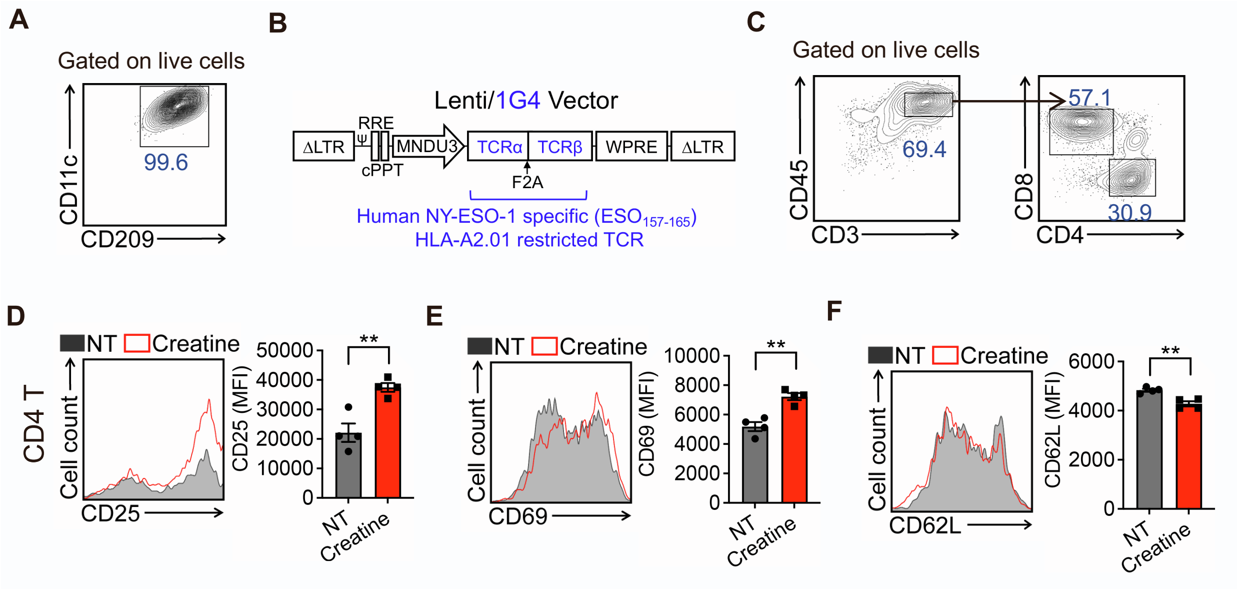

Figure S8. Human MoDC and ESO-T cell generation, related to Figure 6

(A) FACS phenotype of day 5 MoDCs.

(B) Lentivector encoding a human HLA-A2-restricted NY-ESO-1 TCR gene.

(C) Gating strategy for identifying $CD8^{+}$ ESO-T cells from coculture.

(D-F) FACS analysis of CD25 (D), CD69 (E), and CD62L (F) expression in $CD4^{+}$ ESO-T cells (n = 4).

Representative of two experiments. Data are presented as the mean ± SEM. $^{**}p < 0.01$ by Student's t-test.

Table S1. Primers used for quantitative reverse-transcription PCR (RT-qPCR), related to STAR Methods

Gene	Forward Primer 5'-3'	Reverse Primer 5'-3'	Source
Ube2d2	ACAAGGAATTGAATGACCTGGC	CACCCTGATAGGGGCTGTC	Li et al.[1]
CrT	ACTGGGAGGTGACCTTGTGC	CGATCTTTCCTGTTGACTTG	Di Biase et al.[2]
Il1b	TTGACGGACCCCAAAAGAT	GAAGCTGGATGCTCTCATCAG	This paper
Il6	CTGCAAGAGACTTCCATCCAG	AGTGGTATAGACAGGTCTGTTGG	Wang et al.
Tnf	AAGCCTGTAGCCCACGTCGTA	AGGTACAACCCATCGGCTGG	Li et al.[1]
Nos2	CCCTTCAATGGTTGGTACATGG	ACATTGATCTCCGTGACAGCC	This paper
Il12b	CAGAAGCTAACCATCTCCTGGTTTG	TCCGGAGTAATTTGGTGCTTCACAC	This paper
ACTB	GAGCACAGAGCCTCGCCTTT	ACATGCCGGAGCCGTTGTC	Li et al.[1]
CRT	TCCTGGCACTCATCAACAG	ATGAAGCCCTCCACACCTAC	Ellery et al.[3]
IL1B	TCTCAACCCCCAATAAATATAGGA	GATGCCGTCGAGGATGTACC	This paper
IL6	ACTCACCTCTTCAGAACGAATTG	CCATCTTTGGAAGGTTCAGGTTG	This paper
TNF	CTCTTCTGCCTGCTGCACTTTG	ATGGGCTACAGGCTTGTCACTC	Li et al.[1]

SUPPLEMENTAL REFERENCES

1. Li, B., Elsten-Brown, J., Li, M., Zhu, E., Li, Z., Chen, Y., Kang, E., Ma, F., Chiang, J., Li, Y.-R., et al. (2025). Serotonin transporter inhibits antitumor immunity through regulating the intratumoral serotonin axis. Cell *188*, 3823-3842.e21. https://doi.org/10.1016/j.cell.2025.04.032.

2. Di Biase, S., Ma, X., Wang, X., Yu, J., Wang, Y.-C., Smith, D.J., Zhou, Y., Li, Z., Kim, Y.J., Clarke, N., et al. (2019). Creatine uptake regulates CD8 T cell antitumor immunity. J. Exp. Med. *216*, 2869–2882. https://doi.org/10.1084/jem.20182044.

3. Ellery, S.J., Della Gatta, P.A., Bruce, C.R., Kowalski, G.M., Davies-Tuck, M., Mockler, J.C., Murthi, P., Walker, D.W., Snow, R.J., and Dickinson, H. (2017). Creatine biosynthesis and transport by the term human placenta. Placenta *52*, 86–93. https://doi.org/10.1016/j.placenta.2017.02.020.
